# HDAC5 controls a hypothalamic STAT5b-TH axis, the sympathetic activation of ATP-consuming futile cycles and adult-onset obesity in male mice

**DOI:** 10.1016/j.molmet.2024.102033

**Published:** 2024-09-19

**Authors:** Raian E. Contreras, Tim Gruber, Ismael González-García, Sonja C. Schriever, Meri De Angelis, Noemi Mallet, Miriam Bernecker, Beata Legutko, Dhiraj Kabra, Mathias Schmidt, Matthias H. Tschöp, Ruth Gutierrez-Aguilar, Jane Mellor, Cristina García-Cáceres, Paul T. Pfluger

**Affiliations:** 1 Research Unit NeuroBiology of Diabetes, Helmholtz Munich, Neuherberg, Germany; 2 Institute for Diabetes and Obesity, Helmholtz Munich, Neuherberg, Germany; 3 German Center for Diabetes Research (DZD), Neuherberg, Germany; 4 Neurobiology of Diabetes, TUM School of Medicine & Health, Technische Universität München, München, Germany; 5 Van Andel Institute, Grand Rapids, MI, USA; 6 Institute of Experimental Genetics, Helmholtz Munich, Neuherberg, Germany; 7 Biological Research Pharmacology Department, Sun Pharma Advanced Research Company Ltd., Vadodara, India; 8 Neurobiology of Stress Resilience, Max Planck Institute of Psychiatry, Munich, Germany; 9 Division of Metabolic Diseases, TUM School of Medicine & Health, Technical University of München, Munich, Germany; 10 Helmholtz Center Munich, Neuherberg, Germany; 11 División de Investigación, Facultad de Medicina, Universidad Nacional Autónoma de México, Mexico City, Mexico; 12 Laboratorio de Investigación en Enfermedades Metabólicas, Obesidad y Diabetes, Hospital Infantil de México Federico Gomez, Mexico City, Mexico; 13 Department of Biochemistry, University of Oxford, Oxford, UK; 14 Chronos Therapeutics, Oxford, UK; 15 Medical Clinic and Polyclinic IV, Ludwig-Maximilians University of München, Munich, Germany

**Keywords:** Adult-onset obesity, Dopamine, Hypothalamus, Brown fat thermogenesis, Histone deacetylase 5, Futile ATP-consuming cycles

## Abstract

With age, metabolic perturbations accumulate to elevate our obesity burden. While age-onset obesity is mostly driven by a sedentary lifestyle and high calorie intake, genetic and epigenetic factors also play a role. Among these, members of the large histone deacetylase (HDAC) family are of particular importance as key metabolic determinants for healthy ageing, or metabolic dysfunction. Here, we aimed to interrogate the role of class 2 family member HDAC5 in controlling systemic metabolism and age-related obesity under non-obesogenic conditions. Starting at 6 months of age, we observed adult-onset obesity in chow-fed male global HDAC5-KO mice, that was accompanied by marked reductions in adrenergic-stimulated ATP-consuming futile cycles, including BAT activity and UCP1 levels, WAT-lipolysis, skeletal muscle, WAT and liver futile creatine and calcium cycles, and ultimately energy expenditure. Female mice did not differ between genotypes. The lower peripheral sympathetic nervous system (SNS) activity in mature male KO mice was linked to higher dopaminergic neuronal activity within the dorsomedial arcuate nucleus (dmARC) and elevated hypothalamic dopamine levels. Mechanistically, we reveal that hypothalamic HDAC5 acts as co-repressor of STAT5b over the control of *Tyrosine hydroxylase* (TH) gene transactivation, which ultimately orchestrates the activity of dmARH dopaminergic neurons and energy metabolism in male mice under non-obesogenic conditions.

## Highlights

• HDAC5 interacts with STAT5b to co-repress TH gene expression.
• Higher hypothalamic dopamine levels associate with lower sympathetic activity.
• Male, but not female, HDAC5 KO mice fed chow develop obesity due to reduced thermogenesis.
• HDAC5 KO show diminished ATP-consuming futile cycles in BAT, muscle, liver, and WAT.

## Graphical abstract

**Figure:**
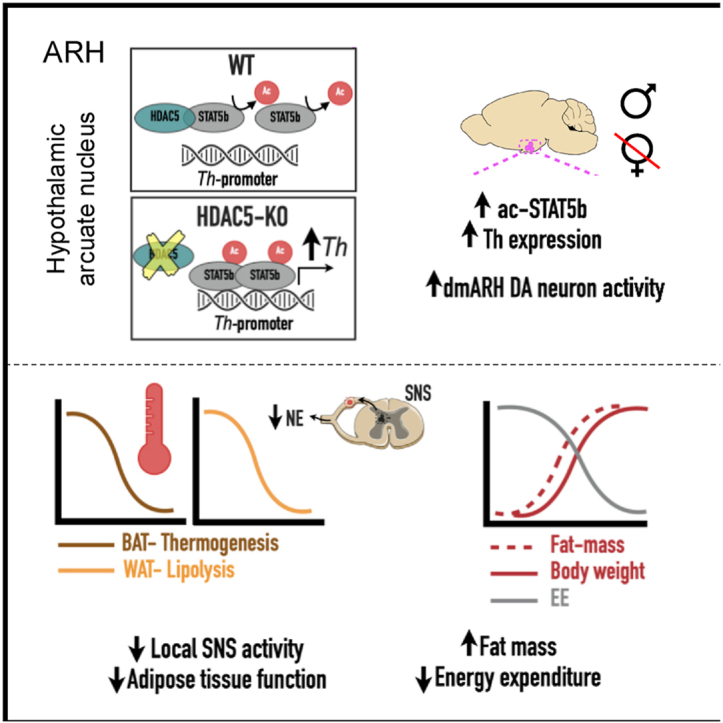

## 1 Introduction

Obesity is a global health problem with the highest prevalence in middle-aged people between 40 and 59 years old (11.5%), followed by the age groups of early adulthood (20–39 years, 9.1%), and late adulthood (>60 years, 5.8%) [1,2]. Modifiable risk factors such as the overabundance of high calorie food, the socioeconomic status and a sedentary lifestyle play a major role in driving age-related obesity. Of similar importance are non-modifiable risk factors such as ageing, genetic and epigenetic traits. Identifying these genetic and epigenetic risk traits, their relative contribution to age-related obesity, and their functional interaction is thus an important task for our ageing society.

Obesity and the concomitant accumulation of metabolic perturbations are reciprocally associated to healthy ageing, with extreme obesity shortening human lifespan by up to 14 years [3]. Similar to aging, obesity has been shown to increase systemic inflammation, promote redox imbalance, mitochondrial dysfunction, and ultimately increase genome instability by shortening telomere length and accelerating the epigenetic age in several tissues [4]. Key determinants for the epigenetic age are processes such as DNA methylation and histone modifications (reviewed by [5,6]), which are catalyzed by enzymes previously linked to both, metabolic control and healthy ageing. Among the most prominent and best studied enzymes is the non-classical histone deacetylase (HDAC) class III family of sirtuins, epigenetic regulators of cellular function that catalyze deacetylation of multiple proteins involved in cellular signaling, transcriptional control and chromatin remodeling [7,8]. When overexpressed in worms, flies, and mice, sirtuin-1 (Sirt1) facilitated healthy ageing and profoundly prolonged lifespan [9]. Consistent with that, we revealed protection from high fat diet (HFD)-induced hepatic steatosis and glucose intolerance in mice with Sirt1 overexpression [10]. Metabolic and longevity benefits were also reported for other HDAC class III family members or their allosteric activator [11,12]. Intense studies are moreover focusing on HDAC class 1, 2, 3 and 4 inhibitors in an effort to develop drugs for healthy aging [13,14].

HDAC5 is a canonical HDAC class 2a family member that has been recently associated with the epigenetic and transcriptional regulation of metabolism [15,16]. It has low enzymatic activity toward histones such as H3 and H4, but functions as scaffold protein to recruit HDAC class I family members to DNA repressor complexes, thereby modifying histone acetylation and DNA-accessibility for transcription factors [17]. We demonstrated that HDAC5 is recruited to the promotor region of interleukin 6 in skeletal muscle cells, where it acts as negative epigenetic regulator of IL-6 synthesis and release. Accordingly, mice globally deficient for HDAC5 showed improved systemic glucose tolerance when subjected to an exercise intervention [16]. Considerably more reports nonetheless focus on the enzymatic activity of HDAC5 toward non-histone proteins, and its impact on cellular functions that go beyond epigenetics [18]. In the past, we revealed that HDAC5 can deacetylate the transcription factor Signal transducer and activator of transcription 3 (STAT3) in hypothalamic neurons, which attenuates both its shuttling between the cytosol and nucleus, and ultimately transactivation of target genes. In young mice with global deletion of HDAC5, the exposure to HFD led to impaired expression of the anorexigenic neuropeptide proopiomelanocortin (POMC) that translated into leptin resistance, elevated food consumption and diet-induced obesity, compared to HFD-fed wildtype controls [15]. When young wildtype and HDAC5 knockout mice were exposed to standard chow diet for 4 months, we did not observe any differences in food intake and body weight, indicating a distinct detrimental effect of HFD exposure on metabolic control in the absence of functional HDAC5 activity.

Here, we aimed to assess whether ageing – similar to HFD feeding – exerts detrimental effects on metabolic control in HDAC5-deficient mice. Specifically, we aimed to assess whether HDAC5 deficiency is linked to an increased propensity for obesity and its sequelae in mature mice, even in the absence of an obesogenic environment. We thus exposed global HDAC5 deficient mice to standard chow diet for up to 7 months and revealed an adult-onset propensity for obesity and comorbid sequelae when compared to wildtype controls. Functional studies unraveled an attenuated adipose tissue lipolysis and thermogenesis, and perturbed dopaminergic signaling in hypothalamic circuits in HDAC5-KO mice that were associated with impaired interaction between HDAC5 and the transcriptional regulator STAT5b in dopaminergic neurons of the dorsomedial arcuate nucleus.

## 2 Methods

### 2.1 HDAC5-KO animal model and body composition

Our study reports sex-dimorphic effects of male and female global HDAC5 KO mice with excision of coding exons 3 to 7 for a lacZ-neomycin resistance cassette that were derived from breeding HDAC5 heterozygous (HDAC5+/−) mice with pure C57BL/6 J background as described previously (Kabra et al., 2016). All mice were group-housed on a 12:12-h light–dark cycle at 23 °C and *ad libitum* and fed on standard chow diet, unless differently indicated. Fat mass and lean mass were measured via whole body Nuclear Magnetic Resonance (NMR) technology (EchoMRI, Houston, TX, USA). All procedures involving animal handling were approved by the committee for Care and Use of Laboratory Animals of the Government of Upper Bavaria, Germany.

### 2.2 Genotyping of mouse lines

Eartags were notched from mice at weaning (21 days old) and DNA was isolated by boiling the eartags for 60 min in 100 μl 50 mM NaOH at 95 °C (ThermoMixer C, Eppendorf). 10 μl 1 M Tris was added to neutralize the reaction. 2 μl of isolated genomic DNA was used for the genotyping PCR (GoTaq® Promega) using protocols and primers described in Supplemental Tables 1 to 3.

### 2.3 Energy homeostasis assessment

Mice were single housed in indirect calorimetry cages (TSE Systems, Bad Homburg, Germany). An acclimation period of 48 h was required for mice to adapt to the new environment. The system was calibrated for a gas reference of 20.9% O2, 0.05% CO2 and 79.05% N2 before starting the measurements. Subsequently, measurements for O2 and CO2 along with energy expenditure, locomotor activity and food and drink intake were recorded every 10 min for 72 h at 24 °C. For cold exposure, the system was similarly calibrated but the cages were acclimatized to 4 °C before mice were individually placed for 5 h with measurements of 10 min per unit of time.

### 2.4 Infrared measurement of temperature in the brown adipose tissue

Mice were individually placed inside a cage under the infrared camera (PI450i, Optris Infrared measurements) which was fixed to a tripod. One video per animal with good visibility of the BAT on an extended position was recorded. Subsequently, the measurement was set for mean value and emissivity = 1.0 on the software Optris PI connect™. The region of interest (ROI) for BAT was defined in the intrascapular region of each mouse.

### 2.5 Necropsy and tissue collection

Prior to sacrifice, mice were food deprived for 6 h and glycemia was measured by sampling blood from the tail vein with a handheld glucometer. Rapidly after cervical dislocation, blood was taken in EDTA-coated syringes and, centrifuged at 2,000×*g* for 10 min at 4 °C to separate the plasma. Rapidly, the skull bone was cut open and the brain lifted. The exposed pituitary gland was extracted from the *sella turcica* and the hypothalamus was dissected from the base of the brain. The prefrontal cortex (PFC) and striatum were thoroughly dissected from coronal slices using anatomical references for guidance. Brown adipose tissue was dissected from the intrascapular region following the characteristic butterfly-shape. Finally, eWAT was dissected from the testis and carefully cleaned from seminal ducts. All samples were snap frozen in liquid nitrogen and stored at −80 °C.

### 2.6 Enzymatic assays

Plasma triglycerides, cholesterol and non-esterified fatty acids were measured by commercial enzymatic colorimetric assay kits (Wako Chemicals, Neuss, Germany). Insulin, leptin, prolactin, GH and TSHb were measured using murine sandwich (capture) ELISA kits (Crystal Chem, 90082; R&D Systems, MOB00; Life Technologies, EMPRL; Merck, EZRMGH-45 K; Biozol diagnostica, USC-CEA463MU-96). All assays were performed according to the manufacturer's instructions. Homeostatic model assessment for insulin resistance (HOMA-IR) was calculated using the formula: HOMA-IR = [fasting serum glucose × fasting serum insulin/22.5] (Turner et al., 1979).

### 2.7 tT4 and tT3 measurement by LC-MS/MS

Thyroid hormones in mouse plasma were analyzed and detected with an UPLC system coupled with a triple quadrupole (QQQ) mass detector. The conditions employed for the elution and quantification were already described for the detection of thyroid hormones in mouse brains (De Angelis et al., 2022). The sample clean-up was performed according to the following protocol: 40–50 μL of mouse plasma was mixed with 60 μL of internal standard (10 pg/μL) and an antioxidant solution (0.15 mL; 25 mg ascorbic acid + 25 mg citric acid + 25 mg dithiothreitol in 1 mL H2O). The mixture was vortexed for 10 s and equilibrated for 1 h at 0 °C. Subsequently, 25 μL of ZnCl2 (2 M in H2O) and 200 μL of CH3OH were added and additionally incubated for 30 min at 0 °C for. Then, the sample was centrifuged at 3000 g for 10 min and supernatant collected. The solid residue was further resuspended in a solution of CH3OH:H2O ((1:1), 0.2 mL), centrifuged, and then extracted as described before. Chloroform (0.6 mL) was added to the combined extracts and the mixture was centrifuged again (3000×*g*, 10 min). The upper level was decanted while the lower phase was re-extracted (CH3OH:H2O 1:1, 0.2 mL). The pooled upper phases were diluted with 1.5 mL pure water. Phosphoric acid was added to reach a final concentration of 2%, followed by the addition of the antioxidant solution (0.2 mL). After vortexing, the mixture was loaded onto a Bond Elut Plexa PCX cartridge, which was preconditioned sequentially with 1.5 mL of pure MeOH and 1.5 mL of water. The cartridge was first washed with 2 mL of 2% formic acid in water and then with 2 mL of MeOH: acetonitrile (1:1, v/v). Analytes were eluted into a vial with 1 mL of 5% ammonium hydroxide in MeOH: acetonitrile (1:1, v/v). The solvent was evaporated and the compounds re-dissolved in 60 μL of a mixture of 20% acetonitrile in water containing 0.1% formic acid for instrumental analysis.

### 2.8 Monoamine measurement by HPLC-ECD

Adipose tissue monoamines were analyzed and detected with a HPLC system coupled with electrochemical detector (ECD) as described previously (Nagler et al., 2018). Briefly, adipose tissue (eWAT, 100–120 mg or BAT 50–70 mg) was homogenized in 200 μL of 0.3 M HClO4 and 4 μL of DHBA (internal standard) by ultrasonication on ice for 30s. Homogenates were centrifuged at 8000×*g* for 10 min. The solution was collected with a 1 mL syringe and a cannula without disrupting the top fat layer. To remove traces of fat, samples were filtered through a 0.2 μm filter (Whatman, P/N 6784-0402). The filtrate was collected in a sample vial and injected into the system.

Brain monoamines were similarly analyzed and detected with HPLC-ECD as described previously (Nagler et al., 2018). Sample clean-up was performed according to the protocol for extraction of monoamines from mouse hypothalamus. Briefly, samples (10–15 mg) were individually homogenized in 200 μl of 0.3 M HClO4 and 4 μL of DHBA 1 ng/μL (internal standard) via ultrasonication (Bandelin Electronics, UW-70) on ice for 30s. Homogenate was centrifuged at 8000×*g* for 10 min at 4 °C and then 20 μL of the supernatant was directly injected into a HPLC-ECD system.

### 2.9 Immunofluorescence in brain slices

Mice were euthanized with CO2 and perfused through the heart using a peristaltic pump. After a washing step with ice-cold PBS, animals were perfused with 4% paraformaldehyde (PFA). Brains were post-fixed overnight in 4% PFA at 4 °C, followed by equilibration with 30% sucrose in Tris-buffered saline (TBS, pH 7.2) for 48 h before sectioning into 30 μm coronal slices using a cryostat (CM3050S; Leica, Germany). Per mouse, three to four brain sections containing the middle portion of the MBH were selected and washed with TBS, incubated in 500 μl of 0.1 M glycine for 30 min with agitation at room temperature and then washed three times with TBS. Subsequently, slices were incubated overnight at 4 °C with primary antibodies (anti-TH, anti-cFOS and anti-NeuN diluted 1:1000) in 500 μl of solution containing 0.25% porcine gelatine and 0.5% Triton X-100 in TBS, pH 7.2. The following day, sections were rinsed three times in TBS, pH 7.2, and incubated with respective secondary antibodies (anti-goat AlexaFluor-647, anti-rabbit AlexaFluor-568 and anti-mouse AlexaFluor-488, diluted 1:5000) diluted in 500 μl of TBS, pH 7.2 containing 0.25% porcine gelatine and 0.5% Triton X-100 for 2 h. Sections were serially washed three times in TBS with the last washing additionally containing DAPI (2 μg/ml in TBS, pH 7.2). Sections were mounted, covered in Elvanol mounting medium (150 mM Tris, 12% Mowiol 4–88, 2% DABCO) and sealed under a coverslip. Acquisition was performed on a Leica SP5.

### 2.10 Fluorescence-activated cell sorting (FACS) for hypothalamic nuclei

Hypothalami were individually processed as previously described by (Krishnaswami et al., 2016) with some modifications to the protocols. Briefly, frozen hypothalami were transferred to a Dounce homogenizer containing 1 mL of freshly prepared ice-cold nuclei isolation buffer (0.25 M sucrose, 25 mM KCl, 5 mM MgCl2, 20 mM Tris pH 8.0, 0.4% IGEPAL 630, 1 mM DTT, 0.15 mM spermine, 0.5 mM spermidine, 1× phosphatase & protease inhibitors, 0.4 units RNasin Plus RNase Inhibitor, 0.2 units SuperAsin RNase inhibitor). Homogenization was achieved by 10 pestle strokes, followed by an incubating on ice for 5 min and 15 more strokes. The homogenate was filtered through a 20 μm cell strainer and centrifuged at 1000×*g* for 10 min at 4 °C. The nuclear pellet was resuspended in 500 μl of staining buffer (PBS, 0.15 mM spermine, 0.5 mM spermidine, 0.4 units RNasin Plus RNase Inhibitor, 0.5% BSA) and incubated for 15 min on ice to allow blocking of unspecific binding. Staining was achieved by adding 1 μl of Anti-NeuN AlexaFlour-488 or IgG-isotype control and incubating for 30 min in dark at 4 °C. Unbound antibody was removed with 1 volume of staining buffer and centrifugation at 1000×*g* for 5 min at 4 °C. Nuclear pellets were resuspended in 1 mL of fresh staining buffer supplemented with DAPI 1 μg/μL (Axio Scope, Zeiss, Germany). Nucleus integrity was assessed in the DAPI channel under a Zeiss microscope (Axio Scope, Zeiss, Germany). Subsequently, samples were sorted in a FACS-Aria III (BD Biosciences) using a 70 μm nozzle. Doublet discrimination and DAPI staining were used for appropriate gating of single nuclei. NeuN- and NeuN + nuclei fractions were defined by the fluorescence intensity in the green channel. Fractions were collected in cold RLT buffer supplemented with DTT, immediately frozen and stored at −80 °C until RNA extraction.

### 2.11 RNA isolation and gene expression analysis

RNA from all sorted samples was isolated using the RNeasy Micro Kit (74004, QIAGEN) and measured with the Agilent RNA 6000 Pico Kit (5067-1513, Agilent) following manufacturer's indications. RNA concentrations were adjusted, and 1 ng RNA was used as input for cDNA synthesis employing the SMART-Seq V4 Ultra® Low Input RNA kit (634888, Takara Bio USA). Gene expression was quantified using TaqMan probes (Supplemental Table 4) in a ViiATM7 Real Time PCR System. Differential gene expression was calculated using the 2-ΔΔCt method normalized to *Malat1*.

For tissue samples, RNA was isolated using the NucleoSpin RNA isolation kit (740955, Machery-Nagel) following manufacturer's instructions. Subsequently, RNA concentrations were measured on a Nanodrop and 1 μg of RNA per sample was reverse-transcribed into cDNA using QuantiTect® Reverse Transcription Kit (205311, QIAGEN). Gene expression was quantified using validated SYBR-green primers or TaqMan probes (Supplemental Tables 4 and 5), in a ViiATM7 Real Time PCR System. Differential gene expression was calculated using the 2-ΔΔCt method normalized to *Hprt*.

### 2.12 High-throughput ChIPmentation

Individual MBH were homogenized as previously described for nuclei isolation for FACS. After centrifugation, the nuclei pellet was thoroughly resuspended in 1 mL of 1% ethanol-free formaldehyde and incubated at RT in agitation for 15 min to allow fixation. Fixation was quenched by adding 60 μl of 2.5 M glycine and further incubated under the same conditions for 5 min. Subsequently, 500 μl of cold PBS were added and nuclei pelleted by centrifugation at 2000×*g* for 10 min at 4 °C. The pellet was resuspended in 200 μl shearing buffer (1% vol/vol SDS, 10 mM EDTA, 50 mM Tris–HCl pH 8, 1 μM trichostatin A, 10 mM nicotinamide, and 1× phosphatase & protease inhibitors) and sonicated for 15 min × 30s ON/30s OFF on high power on a Bioruptor Plus. From this step onwards we followed without modifications the protocol previously described by (Gustafsson et al., 2019). Per sample, we incubated the sheared chromatin with 2 μg of anti-STAT5b or IgG-isotype. To assess STAT5b biding to *Th*, we designed, chose, and validated a pair of primers flanking the genomic sequencing in exon1 containing the described sequence motif for STAT5b (Kanai et al., 2014). For positive and negative control regions we employed the previously described primers for *Pdk4, Socs2* and negative region, respectively (Quagliarini et al., 2019).

### 2.13 Protein extraction

On a mortar filled with liquid nitrogen, adipose tissue (BAT or eWAT) was carefully pulverized. Then 30–40 mg of tissue-powder was weighed and lysed in 400 μl RIPA buffer supplemented with 1% phosphatase and protease Inhibitor Cocktail and 1 mM phenyl-methane-sulfonylfluorid (PMSF) in a Tissue Lyser II for 3 min at 30/sec in pre-cooled racks. Lysates were then cleared by centrifugation at 12,000×*g* for 10 min at 4 °C and supernatants were collected and stored at −80 °C until further processing. For arcuate nucleus, micro-punches were performed in 1 mm fresh brain slices and flash-frozen in liquid nitrogen. ARH micro-punches were lysed in 50 μl of RIPA buffer supplemented with 1% phosphatase and protease Inhibitor Cocktail and 1 mM phenyl-methane-sulfonylfluorid (PMSF) on ice in sonicator with two pulses of 40% potency for 30 s at low power. Lysates were stored at −80 °C until further processing.

### 2.14 SDS-PAGE and western blot

Protein concentrations were measured using BCA protein assay (23225, Thermo Fischer). Concentrations were adjusted to load 10 μg per well, complemented with Laemmli loading buffer plus 5% DTT (sampling buffer), denatured by heating at 95 °C for 5 min and separated on 4–20 % gradient CriterionTM TGXTM Precast Gels. Proteins were transferred to nitrocellulose membranes using a Trans-Blot® Turbo™ (BioRad) set to the mix molecular weight program. Transferred-membranes were blocked with 5% BSA diluted in TBS with 0.1% Tween20 (TBS-T) for 1 h with agitation at RT and incubated with the desired primary antibodies overnight at 4 °C with agitation. The following day, membranes were washed 5 times in TBS-T for 5 min at RT and incubated with the respective HRP-coupled secondary antibodies for 1 h at room temperature with agitation. Washing step was repeated 5 times prior to protein detection with ECL clarity for HRP-induced chemiluminescence inside a ChemiDoc™ Imager (Bio-Rad). When necessary, membranes were stripped with pre-warmed (37 °C) Restore PLUS Western Blot Stripping Buffer for 10 min, blocked for 1 h, and blotted with the desired antibodies. Densitometric analyses of the bands were performed with NIH FIJI software, and arbitrary units were normalized to appropriate controls.

### 2.15 Co-immunoprecipitation assay

Individual hypothalami were transferred to a dounce homogenizer containing 1 mL of freshly prepared ice-cold immunoprecipitation (IP) buffer (50 mM Tris pH 8.0, 1% IGEPAL-630, 150 mM NaCl, 1 mM PMSF, 1 μM trichostatin A, 10 mM nicotinamide, and 1× phosphatase & protease inhibitors). Homogenization was achieved by carefully douncing 20 strokes with the loose pestle, incubating on ice for 10 min and further doucing 30 more strokes with the tight pestle. The homogenate was filtered through a 20 μm cell strainer and incubated for 30 min with agitation at 4 °C. Protein concentration was measured by BCA protein assay and a 10 μg protein aliquot was processed with sampling buffer for loading-input. Per sample, ∼800 μg of protein lysate was incubated with 2.0 μg of antibody (anti-STAT5b or IgG-isotype control) in Lo binding tubes at 4 °C while rotating for 4 h. Each sample halved in two tubes: the first (immunoprecipitation, IP) was left incubating overnight under the same conditions and the second (co-immunoprecipitation, CoIP) was immediately processed. For processing in both cases, 50 μl of μMACS protein-G μBeads were added and incubated for another 30 min at 4 °C with rotation. All further steps were performed at 4 °C and the IP was performed according to the manufacturer's protocol. Proteins were eluted in 40 μl of preheated (95 °C) sampling buffer, separated by SDS-PAGE and transferred to a PVDF membrane for western blotting as described in the previous section. After blocking, the IP membrane and CoIP membranes were first incubated with anti-Lys-acetyl and anti-HDAC5 (1:1000 in blocking buffer) over-night at 4 °C with agitation. The following steps were performed as previously described. After the first detection, membranes were stripped and blotted with anti-STAT5b (1:5000 in blocking buffer) following the same protocol.

### 2.16 Sub-cellular fractioning

Individual hypothalami were homogenized as previously described for nuclei isolation by FACS, with the only difference of reducing the homogenization buffer volume to 700 μl. The homogenate was filtered through a 20 μm cell strainer and centrifuged at 1000×*g* for 10 min at 4 °C. Supernatant was thoroughly collected on a new tube (cytosolic fraction) and the nuclei pellet resuspended in 300 μl of nuclear lysis buffer (50 mM Tris pH 8.0, 1% IGEPAL-630, 0.5 mM sodium deoxycholate, 1 mM PMSF, 1 mM sodium butyrate, 1× phosphatase and protease inhibitors). Protein concentration measurement and subsequent steps were performed as previously described for SDS-PAGE and western blotting. Histone 3 (H3) and GAPDH were used as loading control for the nuclear fractions and cytosolic fractions, respectively.

### 2.17 Statistical analysis

All statistical analyses were performed using GraphPad Prism (V10) or SPSS (V29). All statistical test procedures are indicated in the respective figure legends. Statistically significant outliers in datasets were detected using the Grubbs or Routs tests. Statistically significant differences were considered by p < 0.05, or q < 0.05 in case of multiple t-testing. Data are presented as means ± standard error of the mean (SEM).

## 3 Results

### 3.1 HDAC5-KO male mice developed metabolic dysfunction under non-obesogenic conditions

**Figure 1:**
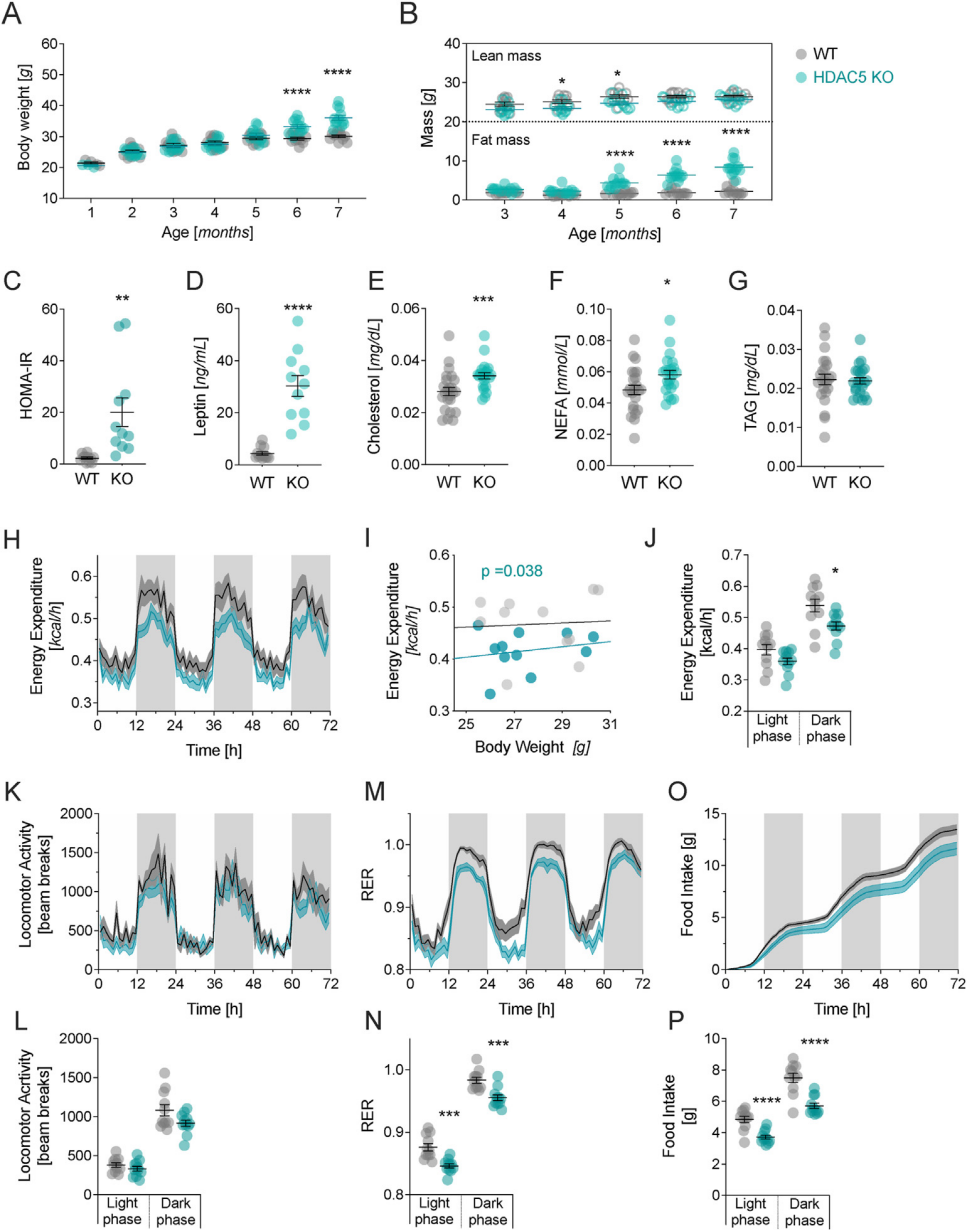
**Chow-fed male HDAC5-KO mice develop adult-onset obesity.** Chow-fed male WT and HDAC5-KO mice were evaluated for changes in (**A**) body weight and (**B**) body composition over a period of 7 months. At 6 months of age, fasting plasma was collected to assess the (**C**) HOMA-IR, (**D**) leptin, (**E**) cholesterol, (**F**) non-esterified free fatty-acids (NEFA) and (**G**) triglyceride (TAG) levels. At 3 months of age, WT and HDAC5-KO males were subjected to 72 h of combined indirect calorimetry to assess (**H**) temporal changes in energy expenditure (EE), (**I**) average total EE values in correlation to body weight, (**J**) average light and dark phase EE, temporal changes and average light and dark phase values for (**K**,**L**) locomotor activity, (**M**,**N**) respiratory exchange ratio (RER) and (**O**,**P**) food intake. Values represent means ± SEM. Statistical analysis were done by either two-way ANOVA with Bonferroni post-hoc test (A,B,K,M,O), two-tailed unpaired Students' t-tests (C-G,J,L,N,P) or ANCOVA with body weight as co-variate (H–J). ∗p < 0.05, ∗∗p < 0.01, ∗∗∗p < 0.001 and ∗∗∗∗p < 0.0001.

We had previously shown an accelerated onset of obesity when 8-10-week-old male global HDAC5 KO mice were exposed to HFD feeding [15]. Here, we reveal that under non-obesogenic conditions, male global HDAC5-KO (*Hdac5*^*−/−*^*)* mice show an age-related increase in body weight starting at 6 months of age (Figure 1A), compared to their wild-type (WT, *Hdac5*^*+/+*^*)* littermates. Interestingly, fat mass was already higher in male HDAC5-KO compared to WT mice at the age of 5 months (Figure 1B). Elevated body weights were confirmed in another cohort of male chow-fed HDAC5 KO mice, with heterozygous HDAC5-KO (*Hdac5*^*+/−*^*)* mice showing an intermediary phenotype (Supplemental Fig. 1A). At the age of 6 months, male HDAC5-KO mice further showed significantly elevated HOMA-IR levels, indicating insulin resistance, as well as elevated plasma leptin, cholesterol and non-esterified free fatty acids (NEFA) levels. Plasma triacylglycerol levels remained unchanged between genotypes (Figure 1C–G).

To identify the underlying physiological mechanism of the age-related increased propensity for adiposity, insulin resistance and dyslipidemia in male HDAC5-KO mice, we performed indirect calorimetry measurements at an age of 3 months, i.e. prior to the onset of weight differences. We revealed that energy expenditure (EE) was reduced in HDAC5 KO compared to WT mice (Figure 1H,I; ANCOVA F (1,19) = 4.993, p = 0.038) after correction for the covariate body weight (F (1,19) = 0.254, p = 0.62). Reduced EE in the light and dark phase (Figure. 1J) was not influenced by locomotor activity, which remained unchanged between genotypes (Figure 1K,L). Rather, we found diminished respiratory exchange ratios (RER) in the HDAC5-KO mice, which points to a switch in nutrient partitioning from carbohydrate to fat burning (Figure 1M,N). Last, we found a reduction in light and dark phase food intake in HDAC5-KO mice, which might be a compensatory response to the diminished energy expenditure (Figure 1O,P).

Shifts in nutrient partitioning and energy expenditure may be based on changes in the endocrine system, for instance via thyroid and adrenal hormones that are known to stimulate basal metabolism, thermogenesis, lipid and glucose metabolism, fat oxidation, and food intake. However, comparable plasma values for thyroid hormones T3 and T4, thyroid stimulating hormone subunit beta (TSHb) as well as for adrenocorticotropin (ACTH) and corticosterone (CORT) in male, 6-months-old WT and HDAC5-KO mice suggest unperturbed functionality for the hypothalamus-pituitary-thyroid (HPT) and hypothalamus-pituitary-adrenal (HPA) axes (Supplemental Fig. 1B-F). Plasma growth hormone (GH) and norepinephrine levels were also comparable between genotypes (Supplemental Fig. 1G,H), but HDAC5-KO mice tended to show lower epinephrine plasma levels (Supplemental Fig. 1I, p = 0.061).

### 3.2 Impaired SNS-driven brown fat thermogenesis in HDAC5-KO mice

**Figure 2:**
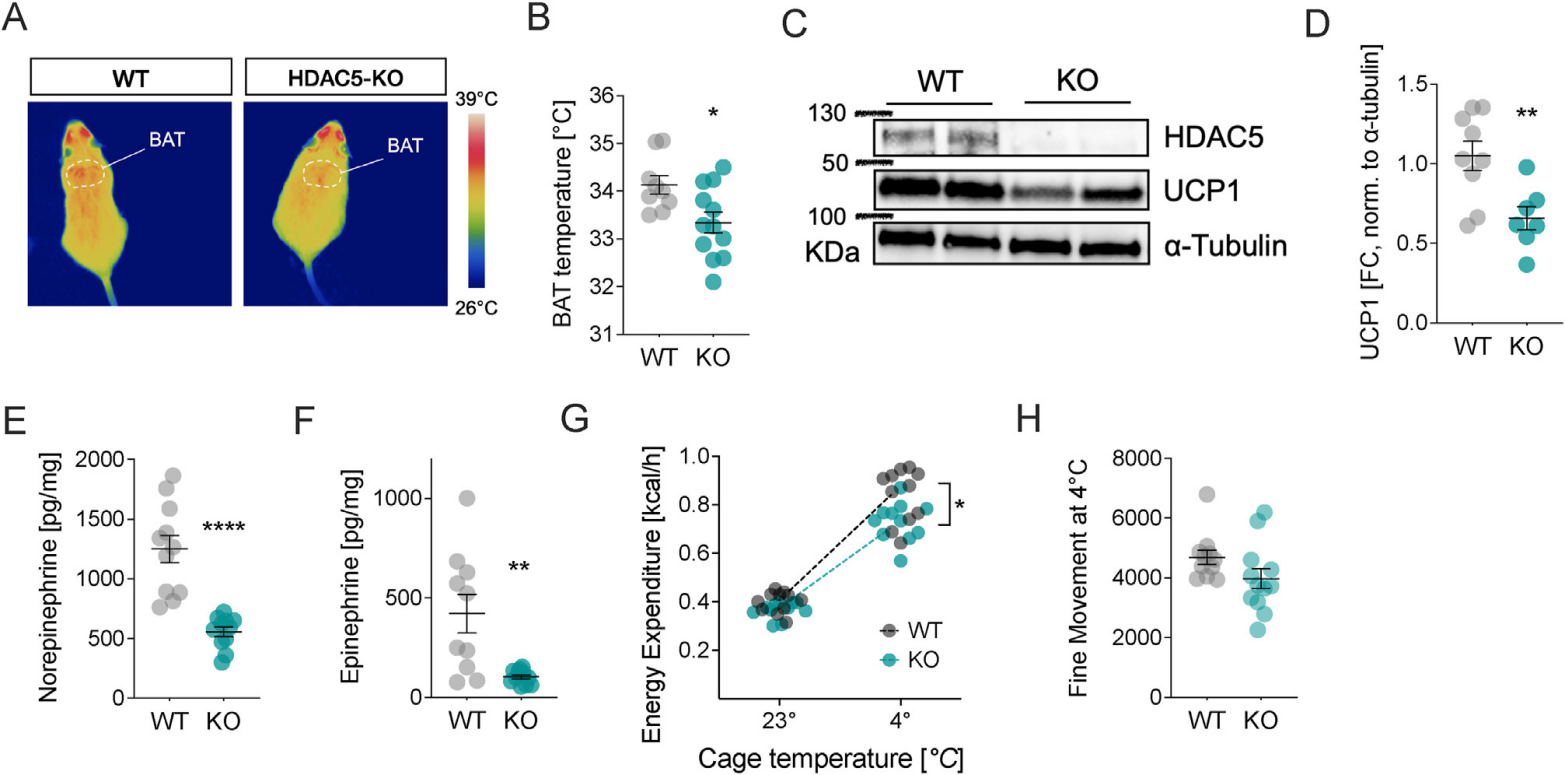
**Impaired brown adipose tissue thermogenesis in male HDAC5-KO mice.** BAT function in male WT and HDAC5-KO mice at the age of 6 months was assessed by (**A**) infrared thermography depicting (**B**) skin temperature above the intrascapular BAT. Subsequent (**C**) Western Blot and (**D**) densitometric analysis of UCP1 protein levels and HPLC-ECD based quantification of (**E**) norepinephrine and (**F**) epinephrine. Additional cohorts of male WT and HDAC-5 KO mice at the age of 3 months were exposed for 5 h to ambient temperature (23 °C) or cold exposure (4 °C) to assess (**G**) average energy expenditure. As surrogate parameter for cold-induced shivering, we moreover recorded the (**H**) cumulative fine movement of mice subjected to 5 h of cold (4 °C). Values represent means ± SEM. Statistical analysis were done by two-tailed unpaired Students' t-test (B–F,H), or two-way ANOVA with Bonferroni post-hoc test (G). ∗p < 0.05, ∗∗p < 0.01 and ∗∗∗∗p < 0.0001. (For interpretation of the references to color in this figure legend, the reader is referred to the Web version of this article).

The tendency for decreased epinephrine prompted us to more systematically assess the status of the sympathetic nervous system (SNS) in the aged and obese HDAC5-KO males on chow diet. Low SNS activity has been long suggested as a risk factor predisposing for weight gain and the development of obesity [19,20]. Particularly the activation of brown adipose tissue (BAT) and induction of thermogenesis by SNS postganglionic nerves seem to be of importance. We thus hypothesized that the decrease in energy expenditure in the HDAC5 KOs is due to a decrease in BAT activity and assessed heat production in BAT under isothermal conditions (23 °C) by means of an infrared camera. We found significantly lower BAT temperatures in 6-month-old HDAC5-KO compared to WT mice (Figure 2A,B). Consistent with the reduced heat production, BAT UCP1 protein levels in HDAC5-KO were reduced by 37% compared to male WT controls (Figure 2C,D). In BAT explants of HDAC5-KO mice, the levels of norepinephrine (NE) and epinephrine (E) were reduced by 48% and 30.3% compared to WT mice, respectively (Figure 2E,F), indicating reduced SNS activity in BAT of HDAC5 KO mice. This was corroborated in HDAC5-KO mice exposed to a cold stimulus (4 °C) that failed to increase energy expenditure to levels recorded for WT mice (Figure. 2G) while fine movement, a surrogate parameter for shivering [21], was comparable between genotypes (Figure. 2H). Together, these observations demonstrate that the absence of HDAC5 is linked with impaired SNS-driven BAT thermogenesis.

### 3.3 HDAC5-KO mice have decreased white adipose tissue lipolysis

**Figure 3:**
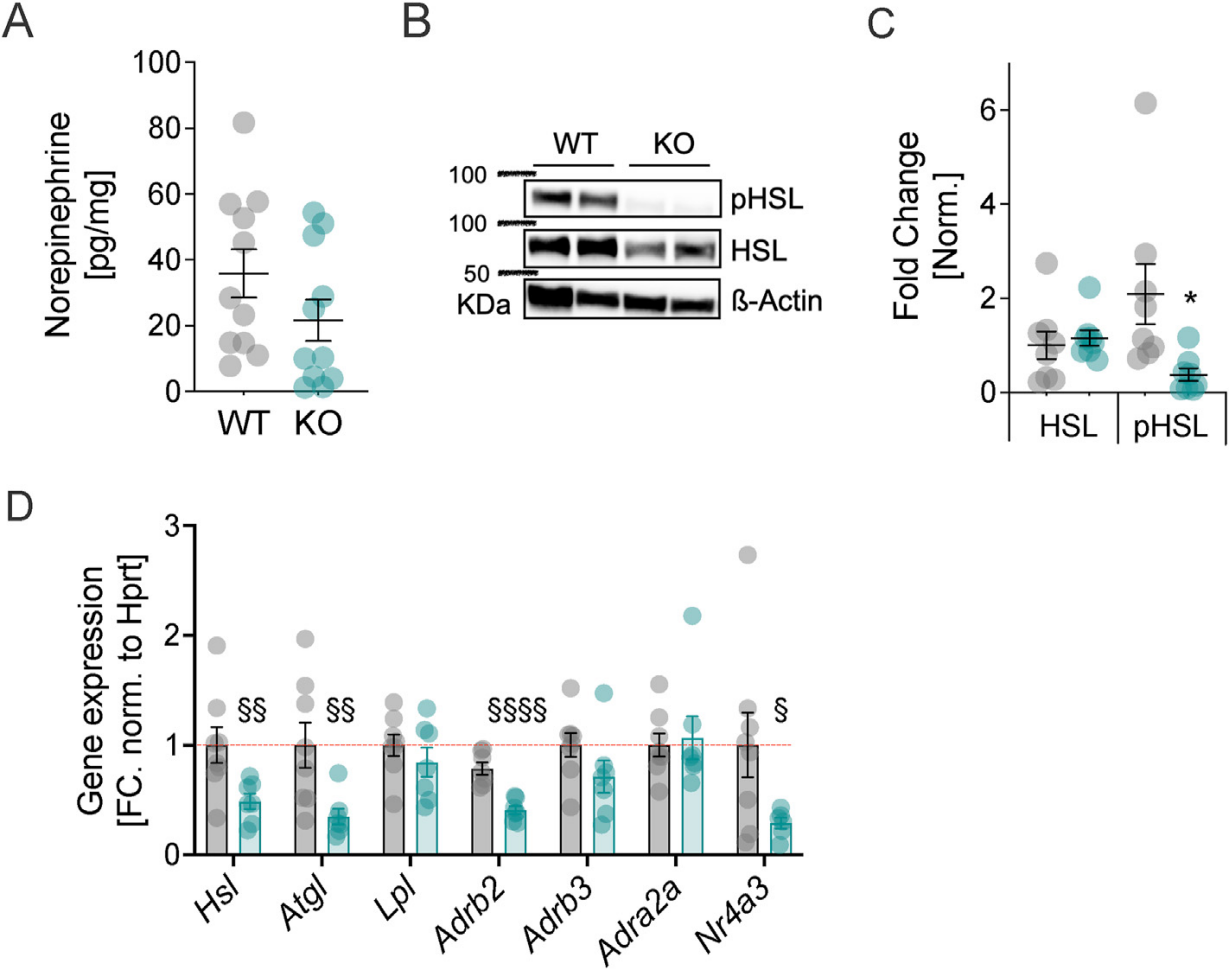
**Impaired adrenergic lipolysis in white adipose tissue of HDAC5-KO males.** Epididymal WAT of HDAC5-KO and WT mice was subjected to (**A**) HPLC-ECD-based detection of norepinephrine levels and (**B**) Western Blotting and (**C**) densitometric analyses for total HSL (normalized to β-actin) and pHSL (normalized to total HSL). (**D**) Quantitative PCR to assess changes in mRNA levels of genes critically involved in adrenergic signaling and lipolysis; hormone-sensitive lipase (*Hsl*), adipocyte triacyl glyceride lipase (*Atgl*), lipoprotein lipase (*Lpl*), beta2-, beta3-and alpha2-adrenergic receptors (*Adrab2, Adrab3,* & *Adra2a*), nuclear receptor subfamily 4 group A member 3 (*Nr4a3*). Values represent means ± SEM. Statistical significance was determined using (A) two-tailed unpaired Students' t-test (A,C) with significance levels indicated as ∗ p < 0.05, ∗∗p < 0.01 and ∗∗∗p < 0.001, or (D) multiple t-testing following the two-stage step-up procedure of Benjamini, Krieger and Yekutieli to control the false discovery rate (FDR). Symbols indicate the following adjusted q-value thresholds: q < 0.1 (^§^), q < 0.05 (^§§^), and q < 0.001 (^§§§§^). The desired FDR threshold was set at 10%.

BAT-induced thermogenesis requires the rapid mobilization of lipid fuel from energy stores in visceral or epidydimal white adipose tissue (eWAT) [22]. This process is initiated by SNS adrenergic stimulation [23], with hormone sensitive lipase (HSL) being the rate limiting lipolytic enzyme [24,25]. Based on our finding of reduced SNS-stimulated thermogenesis in BAT, we first assessed catecholamines by HPLC-ECD and revealed a non-significant trend towards lower NE levels (61% vs. 100%) in eWAT of HDAC5-KO compared to WT (Figure 3A). Next, we tested whether SNS-stimulated lipolysis is perturbed in eWAT of HDAC5-KO mice, and found unaltered HSL protein levels but diminished phosphorylation of HSL at Ser660 in HDAC5-KO mice (Figure 3B,C), indicating reduced enzymatic activity. Last, we found significantly lower gene expression levels for *Hsl, Atgl, Adrab2* and the SNS-sensitive transcription factor *Nr4a3* in eWAT of HDAC5-KO compared to WT mice (Figure. 3D). Expression levels of *Lpl* and the β-adrenergic receptors *Adrab3* and *Adra2a* remained unaltered. In subcutaneous white adipose tissue (scWAT), we found only slightly decreased mRNA levels for the lipolysis regulator *Abhd5* and for *Adra1a* (Supplemental Fig. 2). Together, these results suggest that HDAC5 deficiency decreases SNS stimulation specifically in eWAT leading to reduced HSL activity, and ultimately diminished lipolysis.

### 3.4 HDAC5-KO mice have moderately decreased expression levels of futile calcium and creatine cycle genes

Beside UCP1-driven thermogenesis in brown and beige adipose tissue, other ATP-consuming processes exist such as the futile Ca^2+^ and creatine cycles. We assessed expression changes of key genes of the futile calcium cycle in skeletal muscle, finding only moderate decreases in mitochondrial calcium uniporter (*Mcu*) and ATPase sarcoplasmic/endoplasmic reticulum Ca^2+^ transporting 1 (*Atp2a1*). The latter encodes SERCA1, which is a key enzyme in futile ATP consumption, but also muscle contraction and signaling (Supplemental Fig. 3A). We further saw moderate decreases of the muscle-specific isoform of creatine kinase (*Ckm*), a component of the futile ATP consuming creatine cycle, and alpha-1A adrenergic receptor (*Adra1a*) (Supplemental Fig. 3B). Similar decreases in mRNA levels of futile Ca^2+^ and creatine cycle components, namely *Itpr2* and *Itpr3* as well as *Ckb*, paralleled by decreased levels of adrenergic receptors alpha 1, beta 2 and beta 3, were observed in HDAC5-KO mouse livers, compared to WT controls (Supplemental Fig. 4A,B). We further found diminished mRNA levels for futile Ca^2+^ cycle components *Mcu*, *Itpr1* and *Atp2a2*, but unaltered *Ckb* and moderately elevated *Alpl* expression levels in scWAT of HDAC5-KO mice (Supplemental Fig. 5A,B). In eWAT (Supplemental Figure. 6A,B) and BAT (Supplemental Fig. 7A,B), genes implicated in futile calcium and creatine cycles were comparable between genotypes. Together, these data indicate that futile ATP-consuming calcium and creatine cycles in skeletal muscle, liver and to some extent scWAT could contribute to the overall decrease of thermogenesis in HDAC5-KO mice.

### 3.5 HDAC5-KO mice display elevated hypothalamic dopamine but lower PVH neuronal activity

Attenuated peripheral SNS activities in BAT and eWAT point toward an inactivation of respective CNS regulatory centers, in particular within the hypothalamus. Major players in the hypothalamic control of thermogenesis are monoamines such as dopamine [[26], [27], [28]]. Dopamine-induced hypothermia is a physiological mechanism highly conserved from goldfishes, pigeons, monkeys, rats and mice to humans [27,[29], [30], [31], [32], [33], [34], [35]]. Accordingly, to test whether monoamine signaling is altered in our HDAC5-KO model, we assessed male WT and HDAC5-KO mice for tyrosine hydroxylase (TH), a marker for dopaminergic neurons and cFOS, a marker for neuronal activity.

**Figure 4:**
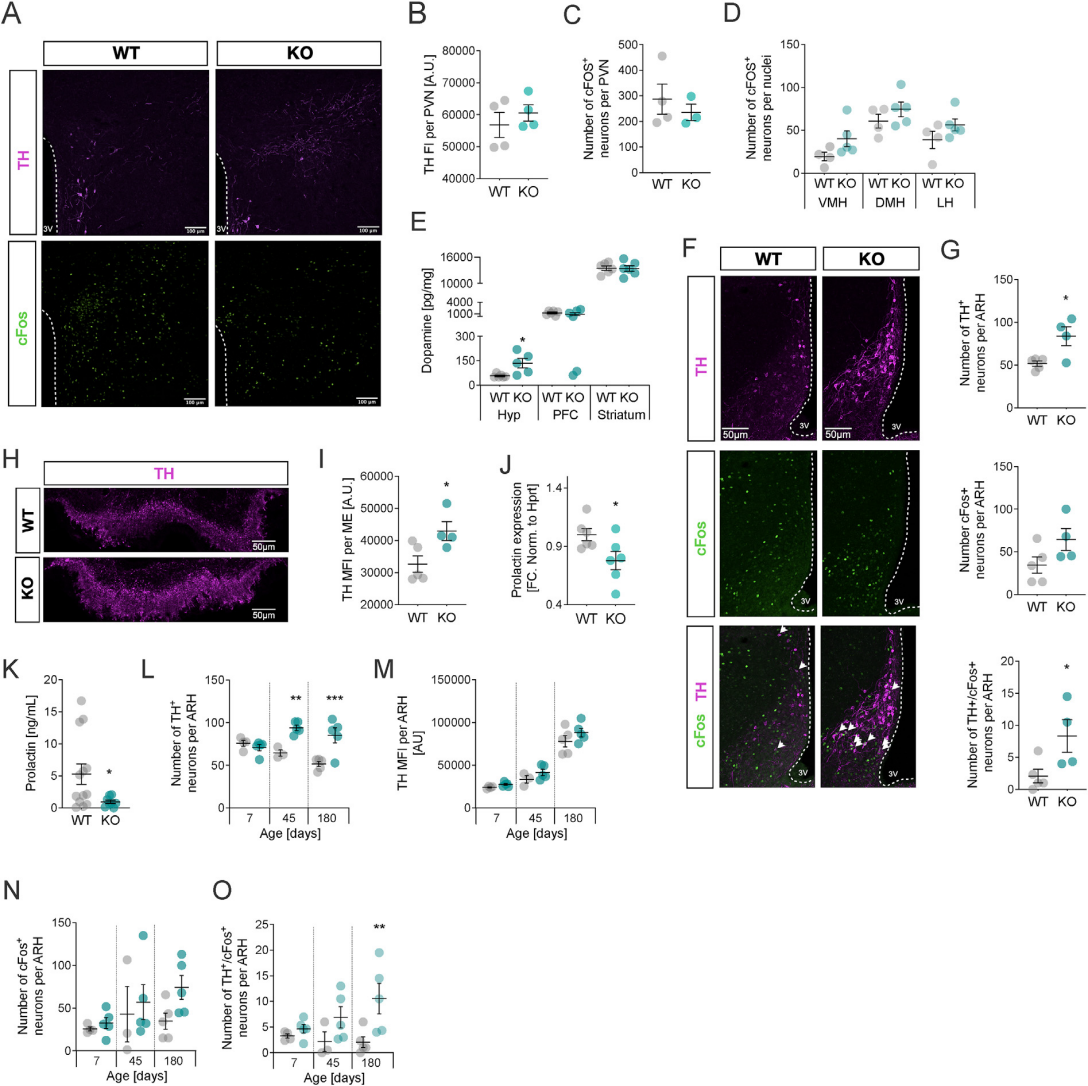
**Persistent activation of dopaminergic neurons in the dorsomedial ARH of HDAC5-KO males drives hypothalamic dopamine levels.** (**A**) Co-immunostainings for TH and cFOS in the paraventricular hypothalamus (PVH) from HDAC5-KO and WT mice were assessed for (**B**) tyrosine hydroxylase (TH) fluorescence intensities (FI) and numbers of cFOS + neurons within the (**C**) PVN or (**D**) additional areas such as the ventromedial (VMH, dorsomedial (DMH) or lateral hypothalamus (LH). (**E**) Dopamine levels were assessed by HPLC-ECD in the hypothalamus (Hyp), prefrontal cortex (PFC) and striatum. (**F**) Co-immunostainings for TH and cFOS in the hypothalamic arcuate nucleus (dmARH) revealed elevated numbers of (**G**) TH, cFOS and TH-cFOS double-positive neurons. (**H**) TH immunostaining and (**I**) fluorescence intensity in the median eminence (ME). Prolactin (**J**) mRNA levels in the pituitary and (**K**) plasma concentrations. (**L-O**) TH and cFOS immunostainings in 7, 45 and 180 days-old WT and HDAC5 KO males were assessed for (**L**) the number of TH + neurons, (**M**) the TH mean fluorescence intensity (MFI), (**N**) the number of cFOS + neurons and (**O**) the number of TH-cFOS double-positive neurons. Values represent means ± SEM. Statistical analysis were done by two-tailed unpaired Students' t-tests. ∗p < 0.05, ∗∗p < 0.01, ∗∗∗p < 0.001 and ∗∗∗∗p < 0.0001.

We first focused on the paraventricular hypothalamus (PVH), a major nucleus for the control of the sympathetic tone, BAT thermogenesis and energy expenditure [36], but found unaltered TH protein (Figure 4A,B) or cFOS^+^ neurons in HDAC5-KO and WT mice (Figure. 4C). Likewise, we found comparable levels of cFOS-positive nuclei in the ventromedial, dorsomedial and lateral hypothalamus (VMH, DMH, LH; Figure. 4D).

Next, we used HPLC-ECD to interrogate hypothalamic dopamine concentrations of HDAC5-KO mice and found a significant 2.4-fold increase compared to WT mice (Figure. 4E). Dopamine measurements in the prefrontral cortex (PFC) and striatum, two alternative regions rich in dopaminergic neurotransmission, did not reveal differences between genotypes. Our data thus suggest a specific and local activation of dopamine signaling in the hypothalamus upon HDAC5-deletion.

### 3.6 Persistent activation of dopaminergic neurons in the dorsomedial ARH of HDAC5 KO mice

In order to identify the source of the increased hypothalamic dopamine levels in HDAC5 KO males, we next focused on the dopaminergic neurons in the dorsomedial ARH (dmARH) that constitute a major source of dopamine for the control of our endocrine system as well as energy homeostasis. Historically, dmARH dopaminergic neurons were considered a homogenous population denoted tuberoinfundibular dopaminergic (TIDA) neurons that release dopamine from the pre-synaptic terminals onto median eminence (ME) to inhibit prolactin secretion in the pituitary [37,38]. More recent, dmARH proved to be a heterogeneous population, not only projecting to the ME, but locally to melanocortin neurons and other unidentified neurons in the PVH [36,39]. Accordingly, co-staining for TH and cFOS in coronal sections containing the ARH revealed a 1.6-fold increase in the number of dmARH TH^+^ neurons, 1.9-fold elevation in the number of dmARH cFOS^+^ and 4-fold increase in co-localized dmARH TH^+^cFOS^+^ neurons in HDAC5-KO compared to WT mice. (Figure 4F,G). Likewise, quantification of TH immunoreactivity in the ME unearthed 1.3-fold higher mean fluorescence intensities in HDAC5-KO compared to WT mice (Figure 4H,I). Diminished prolactin (*Prl)* gene expression levels in the pituitary (Figure. 4J) and a largely abolished secretion into circulation (Figure. 4K) are further in line with a chronic over-activation of dmARH dopaminergic neurons and elevated hypothalamic dopamine concentrations in HDAC5-KO mice of 6 months of age.

### 3.7 Effects of HDAC5 deficiency on dmARH TH + neurons are absent in neonates, and females

In neonates, dmARH dopaminergic neurons require endogenous prolactin to undergo differentiation and maintenance [40,41]. Prompted by the near absence of prolactin in adult male HDAC5 deficient mice, we next assessed the number of TH^+^ neurons in neonates (7 days old), but found no differences between genotypes (Figure. 4L). In contrast, young (45 days old) and adult (180 days old) mice had significantly higher numbers of TH-positive dmARH dopaminergic neurons. TH levels, quantified as mean fluorescence intensities, and the number of cFOS^+^ neurons were comparable for both genotypes, respectively (Figure 4M,N). However, in mice aged 180 days, we found that higher numbers of TH^+^ neurons correlated with increased neuronal activation, evidenced by TH^+^- cFOS^+^ co-staining (Figure. 4O).

**Figure 5:**
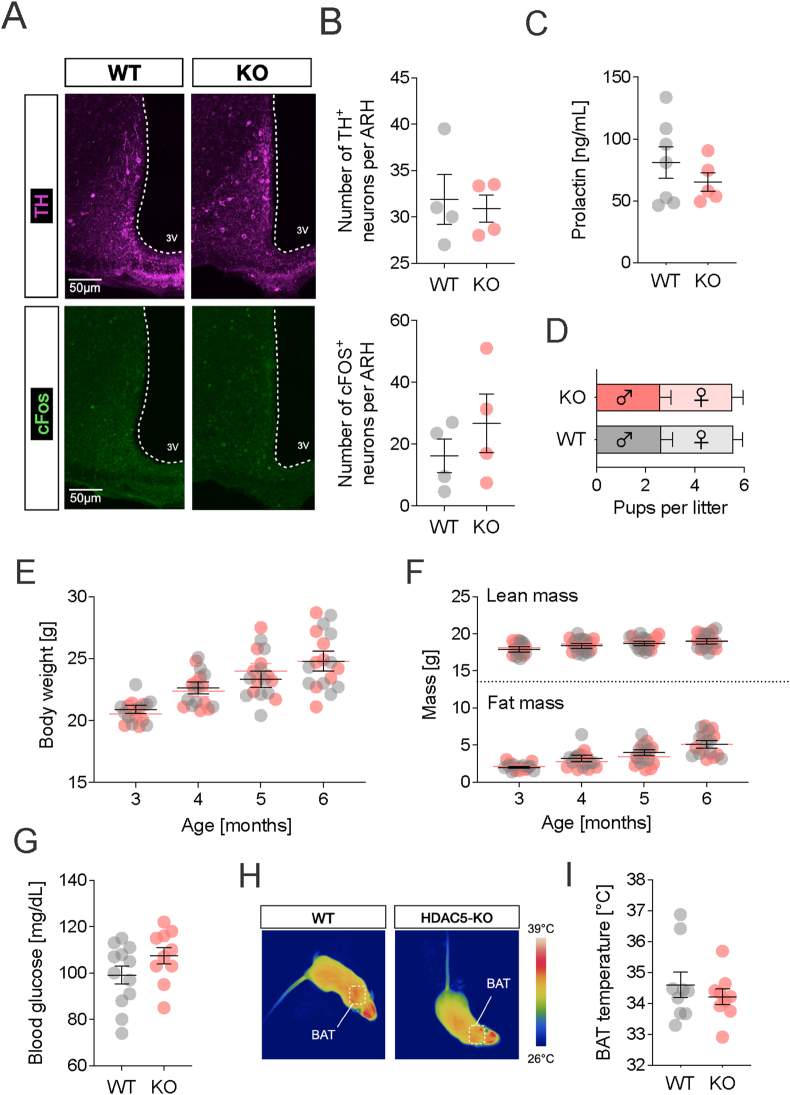
**Unperturbed hypothalamic dopamine tone, systemic metabolism and BAT thermogenesis in HDAC5-KO females.** (**A**) Co-immunostainings for TH and cFOS in the hypothalamic arcuate nucleus (ARH) of HDAC5-KO and WT female mice were evaluated for the numbers of (**B**) tyrosine hydroxylase (TH) and cFOS + neurons. (**C**) Plasma levels of prolactin. (**D**) Average number of pups and sex distribution per litter. (**E**) Body weight and (**F**) body composition over a period of 6 months. (**G**) fasting blood glucose levels and (**H**) infrared thermographic images depicting heat production with the respective quantification of (**I**) surface skin temperature above the intrascapular BAT region. Values represent means ± SEM. Statistical analysis were done by two-tailed unpaired Students' t-tests (B,C,G,I) or two-way ANOVA with Bonferroni post-hoc test (D–F).

Adult female chow-fed WT and HDAC5-KO mice had comparable numbers of TH^+^ dmARH and cFOS positive neurons in the ARH (Figure 5A,B). Likewise, they did not differ in their circulating prolactin levels (Figure. 5C), or in fertility, evidenced by equal litter sizes and sex ratios (Figure. 5D). Last, female HDAC5-KO mice did not show any of the pathologic features observed in their male KO counterparts. Female KOs neither differed in body weight (Figure. 5E), body composition (Figure. 5F), fasting blood glucose (Figure. 5G), nor in BAT thermogenesis (Figure 5H,I) compared to WT females.

Overall, these results demonstrate an impact of HDAC5 deficiency on TH + dmARH neurons already in young male HDAC5-KO mice that persists through adulthood, but unperturbed neonatal dmARH TH^+^ neuronal differentiation and maintenance. The data furthermore reveal sexual dimorphism, as only male HDAC5-KO show an increased propensity for chronic dmARH dopaminergic overactivation and age-related obesity.

### 3.8 Hypothalamic HDAC5 interacts with STAT5b to control TH transactivation

**Figure 6:**
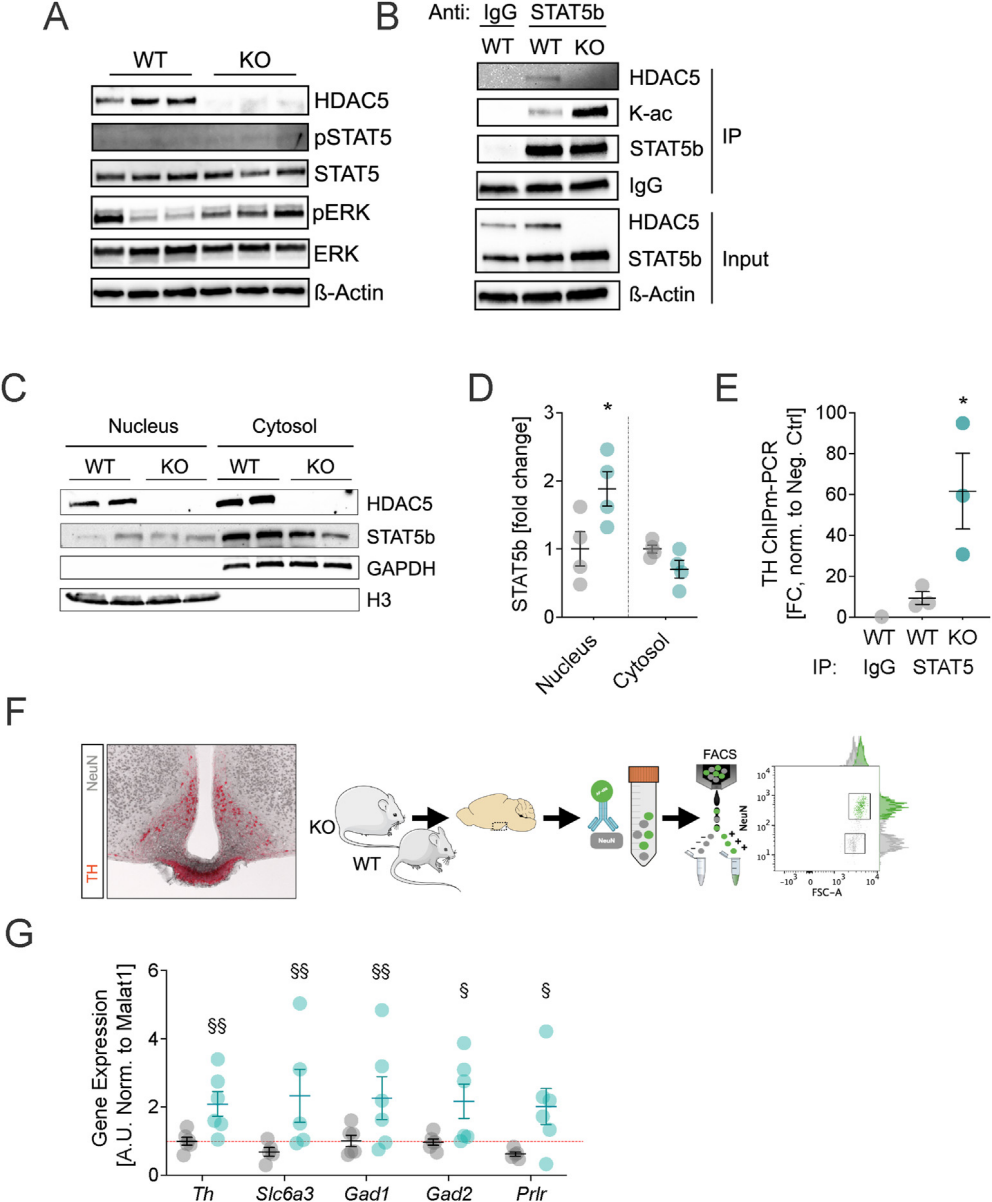
**HDAC5 interacts with STAT5b to drive its nuclear translocation and *Th* transactivation.** Micropunches from the ARH region of male WT and HDAC5-KO mice were subjected to (**A**) Western Blotting for HDAC5 (total) and STAT5b and ERK (total & phospho: p), or (**B**) co-immunoprecipitation using anti-STAT5b and Western blotting detection of HDAC5, lysin acetylation (K–Ac), Stat5b, IgG or beta-actin. (**C**) Western blotting of nuclear and cytosolic fractions depicting the subcellular locations of HDAC5 and STAT5b, with (**D**) densitometric analyses for STAT5b normalized to the housekeeping proteins H3 in the nucleus and GAPDH in the cytosol of male WT and HDAC5-KO hypothalami. (**E**) Following high throughput ChIPmentation with an anti-STAT5 antibody, binding of STAT5 to the promotor region of Th was assessed by qPCR. (F,G) Expression levels of the STAT5 target genes *Th* and *Prlr*, as well as neurotransmitter-linked *Slc6a3*, *Gad1* and *Gad2* in TH expressing cells of the ARH of WT and HDAC5-KO males were assessed by the (**F**) selective enrichment of TH+/NeuN- cells using FACS, and (**G**) qPCR with Malat1 as housekeeping gene. Values represent means ± SEM. Statistical analysis were done by (D, E) Students' t-tests (∗p < 0.05) or (G) multiple t-testing following the two-stage step-up procedure of Benjamini, Krieger and Yekutieli and a FDR of 10% (^§^q < 0.1, ^§§^q < 0.5).

The activation of dmARH dopaminergic neurons has historically been attributed to the activation of STAT5b, ERK1/2 and consequently TH [[42], [43], [44]]. We applied Western Blot analyses to ARH micro punches of adult male WT and HDAC5-KO mice, but found no differences in total protein as well as phosphorylation levels of STAT5b and ERK1/2 (Figure 6A). STAT5b can further be the subject of acetylation as alternative mode of activation [45,46]. Co-immunoprecipitation of STAT5 from hypothalamic lysates of WT and HDAC5-KO mice indeed revealed higher levels of total lysine (K) acetylation when HDAC5 was absent (Figure. 6B). In the past, we had shown that HDAC5 can regulate STAT3 acetylation, which induces sequestration of STAT3 to the nucleus [15]. Accordingly, to assess the impact of HDAC5 on STAT5 localization, we separated hypothalamic lysates into nuclear and cytosolic fractions, and found preferential enrichment of STAT5b in the nucleus of HDAC5-KO but not WT mice (Figure. 6C,D). To confirm that HDAC5 deficiency is not only linked to elevated STAT5 acetylation and nuclear translocation, but also to higher transactivation of gene expression, we next screened the sequence of *Th* for a STAT5b consensus binding motif which we located approximately 40 bp downstream of the start codon of exon 1. Next, we applied high-throughput ChIPmentation-qPCR, and identified significantly enhanced recruitment of STAT5b to *Th* exon1 in HDAC5-KO compared to WT hypothalamic samples (Figure. 6E). Last, to confirm elevated *Th* expression in dopaminergic dmARH neurons of HDAC5-KO mice, we applied fluorescence-activated cell sorting (FACS) to selectively collect these nuclei by applying NeuN as negative selection marker since TH neurons in the dmARH did not express NeuN in co-imunohistochemistry stainings (Figure. 6F). Consistent with our ChIPm-qPCR result of higher STAT5b occupation at the *Th* promoter, we found a significant up-regulation of *Th* gene expression in NeuN-negative dmARH nuclei of HDAC5-KO compared to WT mice (Figure. 6G), accompanied by increased mRNA levels of related genes with significant roles in neurotransmitter synthesis, transport, and signaling, namely the dopamine transporter *(Slc6a3)*, glutamate decarboxylases 1 and 2 (*Gad1 & Gad2)* and the known STAT5 target prolactin receptor (*Prlr*). Overall, our data suggest that HDAC5 interaction with STAT5b in the hypothalamus is necessary for appropriate STAT5b subcellular localization and transcriptional activity.

## 4 Discussion

Epigenetics mechanisms are an important component of healthy aging. Here, we describe a functional role for the epigenetic regulator HDAC5 in the control of age-related obesity under non-obesogenic conditions. We were able to show that male global HDAC5-KO mice develop adult-onset obesity starting at 6 months of age, driven by marked reductions in adrenergic-stimulated, ATP-consuming processes such as BAT-thermogenesis, skeletal muscle, WAT and liver calcium and creatine futile cycles as well as WAT-lipolysis and ultimately energy expenditure. Loss of HDAC5 was linked to higher dopaminergic neuronal activity within the dorsomedial arcuate nucleus (dmARC) and elevated hypothalamic dopamine levels. Mechanistically, we reveal that hypothalamic HDAC5 acts as co-repressor of STAT5b to control *Th* transactivation, which ultimately orchestrates the activity of dmARH dopaminergic neurons and energy metabolism in male mice under non-obesogenic conditions.

Obesity in humans is subcategorized as childhood-adolescence (<20 years old) or adulthood obesity (≥20 years old) [47,48]. Mature adult mice ranging from 3 to 6 months of age would thereby represent young human adults ranging from 20 to 30 years old [49], while mice with an age of 10–14 months would correspond to middle aged humans of 38–47 years (Flurkey et al., 2007). Mature-onset obesity has been reported for various murine knockout models, e.g. for IL-1R, IL-6, p62, TLR2 and OTR [[50], [51], [52], [53]], and is now also linked with HDAC5 deficiency. Of note, the obese phenotype of HDAC5 KOs occurs under non-obesogenic conditions, and is accompanied by hyperleptinemia, glucose intolerance and dyslipidemia, all of which are typical hallmarks for the metabolic syndrome.

The phenotype of obesity and metabolic syndrome observed in our mouse model closely mirrors human genomic studies that demonstrate associations of HDAC5 with whole body fat mass [54], height [55,56], HDL cholesterol levels [57], apolipoprotein A1 levels [57], and diastolic blood pressure [58]. Summary statistics were downloaded from the NHGRI-EBI GWAS Catalog [59] on 25/08/2024 (Supplemental Excel File). Likewise, two independent statistical approaches, the Human Genetic Evidence Score [60] and the Polygenic Priority Score [61], predicted HDAC5 as causal gene for obesity and altered lipid metabolism (Supplemental Excel File and Supplemental Figure 8).

Mechanistically, mature adult-onset obesity is either the result of continuously higher calorie intake, or decreased energy expenditure. In HDAC5-KO male mice, assessment of energy homeostasis by indirect calorimetry unveiled an overall decrease in energy expenditure. Our murine data are consistent with several longitudinal studies performed in southwestern American Indians in which the high prevalence of obesity in young adults was accompanied by low metabolic rate, spontaneous physical activity, fat-oxidation and SNS activity [20,[62], [63], [64]]. In line with that, we observed diminished thermogenesis and lipolytic capacity, lower UCP1 protein expression and blunted HSL activation in BAT and WAT from HDAC5-KO compared to WT mice, respectively. Reduced norepinephrine levels in both BAT and eWAT indicated a blunted local SNS activity upon HDAC5 deletion. In humans, BAT thermogenesis is inversely correlated with BMI and fat-mass when comparing overweight and obese to normal weight patients, and in healthy men throughout winter and summer seasons [65,66]. Perturbed mobilization of energy stores by lipolysis was also documented in obese patients, whose adrenergic-induced lipolysis was blunted [[67], [68], [69]]. Lower mRNA levels of adrenergic receptors and key components of ATP-consuming futile calcium and creatine cycles were further consistent with diminished thermogenesis from liver, WAT and especially skeletal muscle, which profoundly contribute to systemic energy expenditure [70]. Such effects could be tissue-autonomous, given the widespread HDAC5 expression and earlier reports on direct perturbations of glucose and lipid metabolism in hepatocytes and myotubes deficient for HDAC5 [16,71]. Our compiled data alternatively advocates for an impaired CNS (control) - WAT/BAT/liver/muscle (supply/thermogenic) axis that promotes the decrease in energy metabolism and weight gain. However, causality remains to be established, which is a limitation that warrants future studies. These studies, potentially under thermoneutral conditions, should aim to delineate both regulatory components, i.e. the central control vs. tissue-autonomous effects, and further assess whether diminished BAT thermogenesis could be an initial driver for subsequent decreases in lipolysis in eWAT and scWAT and futile calcium and/or creatine cycles in muscle, liver and scWAT. Such studies should moreover address the still unresolved and controversially discussed question whether ATP-consuming futile cycles, including BAT thermogenesis, significantly contribute to human energy expenditure and weight control.

Attenuated local adrenergic tone, probably due to perturbed SNS activity, in HDAC5-KO mice was accompanied by the persistent activation of dopaminergic neurons in the dorsomedial ARH of HDAC5-KO when compared to WT mice. In contrast to striatal increases in dopamine, which are strongly associated with food reward and hedonic overeating [[72], [73], [74], [75]], dopamine signaling in the hypothalamus has rather been implicated with the regulation of autonomic functions, including SNS activity. As such, recent reports showed intrahypothalamic inhibitory dopaminergic projections connecting the ARH to the PVH [36]. Moreover, centrally acting dopamine D2 receptor selective agonists like quinpirole and 7-OH-DPAT reduced sympathetic outflow during cold-induced BAT-thermogenesis in rodents [76]. Consistent with these reports, we found increased hypothalamic dopamine concentrations and activated dmARH dopaminergic neurons in obese HDAC5-KO mice. Notably, HDAC5-driven obesity in mature mice was not associated with increased food intake, nor with changes in dopamine levels in reward-associated brain areas such as the striatum, which both constitute classical hallmarks of diet-induced obesity mouse models [[77], [78], [79]]. Thus, these findings suggest that loss of HDAC5 drives late-onset perturbations in energy balance via separate, reward-independent mechanisms that rather include an attenuated thermogenic drive due to overactivation of dmARH neurons. The exacerbated TH^+^ immunoreactivity in the ME and inhibition of prolactin expression and secretion from the pituitary provide additional physiological proof for the persistent overactivation of dmARH neurons in the tuberoinfundibular pathway in HDAC5-KO compared to WT mice. Next to the dmARH, the lateral hypothalamus (LH) and the adjacent zona incerta were also linked with a D2R-driven yet food intake-independent stimulation of BAT thermogenesis (Folgueira et al., 2019). Comparable levels of cFOS in the LH in our HDAC5 WT and KO males nonetheless argue against a role for HDAC5 in these D2R neurons.

Whether decreased circulating prolactin levels observed in our male HDAC-5 KO mice contribute to their metabolic dysfunction remains unclear. Hyperprolactinemia has been historically associated with metabolically deleterious effects, such as predispositions to brown fat whitening, obesity and the metabolic syndrome [[80], [81], [82]]. Contrasting those reports, in middle-aged and elderly male patients lower circulating prolactin levels were associated with the metabolic syndrome, major cardiovascular events and sexual dysfunction [83,84]. Metabolic perturbations by low prolactin levels were later confirmed by large cohort clinical studies, and benefits of elevated prolactin were reported for adipose tissue function and insulin sensitivity [85,86]. Likewise, metabolically healthy obese subjects were shown to have elevated circulating prolactin levels [87]. Those reports, associating metabolic perturbations to both elevated as well as diminished prolactin levels, are finally contrasted by weight loss as well as dopamine agonist treatment studies that - despite restoring metabolic fitness and BMI - found no association between prolactin and metabolic parameters in male rodents and humans [88].

Collectively, our data are in line with reports associating low prolactin levels with metabolic perturbations, but we are unable to provide evidence that low prolactin levels in HDAC5-KO mice will also exert deleterious metabolic effects. Since prolactin secretion is tightly controlled by a dopaminergic negative feedback loop, our data are merely pointing to a, potentially prolactin-independent, over-activation of dmARH dopaminergic neurons which induced a hypothalamic inhibitory dopaminergic tone in male HDAC5-KO compared to WT mice.

DmARH dopaminergic neuron activation exhibits significant plasticity and complexity in female compared to male mice. During late pregnancy and lactation, elevated prolactin levels are essential for supporting lactation and maternal behavior, accompanied by the suppression of UCP1-driven BAT thermogenesis to conserve energy [89]. The contrary, a relative prolactin deficiency, does however not seem to increase UCP1 expression, at least in our male KO mice. In females, high prolactin levels or a lack of hypothalamic prolactin receptors can cause infertility [90]. Under these energy-demanding conditions, prolactin-controlling dmARH dopaminergic neurons shift from dopamine-mediated inhibition to met-enkephalin-mediated stimulation of the pituitary [43]. Our results showed comparable numbers and activation of dmARH dopaminergic neurons, plasma prolactin concentrations and fertility between genotypes in female mice. Consistently, BAT-thermogenesis, body composition and ultimately body weight remained unperturbed in HDAC5-KO compared to WT females. Due to the plastic phenotype of dmARH dopaminergic neurons in females, HDAC5 might not play a similar role in both sexes. Deciphering the exact mechanisms for this sexual dimorphism in the activation of dmARH dopaminergic neurons in HDAC5-KO mice escaped our present research but warrants future studies.

Last, we decipher the molecular basis for dopaminergic over-activation in dmARH neurons by revealing a STAT5b acetylation-depended mechanism controlled by hypothalamic HDAC5. Although *Th* transcriptional regulation had been attributed to STAT5b, evidence for its binding to the *Th* promotor was lacking [43,[91], [92], [93]]. Prompted by previous *in vitro* reports on an acetylation-depend homodimerization and activation of STAT5b upon prolactin stimulation, which could be reversed by HDAC6, HDAC3, SIRT1 or SIRT2 overexpression in human breast T47D cells [46], we interrogated a role for HDAC5 in STAT5b-driven *Th* transactivation. Herein, we showed *ex vivo* in hypothalamic murine samples an acetylation-dependent nuclear translocation of STAT5b, the recruitment of STATB5 to *Th*, and higher *Th* gene expression. This mechanism was exacerbated in HDAC5-KO mice. This data adds further evidence for a function of HDAC5 as transcriptional co-repressor in the brain, similar to its interaction with transcription factors such as MEF2, SRF or CREB [[94], [95], [96]]. Specifically, we reveal a HDAC5-mediated transcriptional repression of *Th* via deacetylation of STAT5b, which is necessary for limiting the activation of dmARH dopaminergic neurons in male mice.

In sum, our study suggests a novel functional role for hypothalamic HDAC5 as co-repressor of STAT5b that orchestrates the activity of dmARH dopaminergic neurons and ultimately the hypothalamus-controlled adrenergic-stimulation of BAT-thermogenesis, WAT-lipolysis and skeletal muscle and liver futile calcium and creatine cycles. This mechanism was restricted to male mice, age-dependent, and associated with diminished local sympathetic activation as well as lower circulating epinephrine levels. In consequence, male mice with a global deficiency for HDAC5 had lower energy expenditure, and accumulated more fat mass starting at 5 months of age. Accordingly, our work reveals the importance of HDAC5 for appropriate metabolic homeostasis in mature, adult male mice.

## CRediT authorship contribution statement

**Raian E. Contreras:** Writing – review & editing, Writing – original draft, Methodology, Investigation, Formal analysis, Conceptualization. **Tim Gruber:** Methodology, Investigation. **Ismael González-García:** Writing – review & editing, Methodology, Investigation. **Sonja C. Schriever:** Writing – review & editing, Validation, Supervision, Project administration, Methodology, Investigation. **Meri De Angelis:** Writing – review & editing, Methodology, Investigation. **Noemi Mallet:** Formal analysis, Investigation, Methodology. **Miriam Bernecker:** Writing – review & editing, Methodology, Investigation. **Beata Legutko:** Methodology, Investigation. **Dhiraj Kabra:** Writing – review & editing, Resources, Conceptualization. **Mathias Schmidt:** Methodology, Investigation. **Matthias H. Tschöp:** Resources. **Ruth Gutierrez-Aguilar:** Formal analysis, Investigation, Methodology, Visualization. **Jane Mellor:** Writing – review & editing, Validation, Supervision, Resources. **Cristina García-Cáceres:** Supervision, Resources, Methodology. **Paul T. Pfluger:** Writing – review & editing, Writing – original draft, Visualization, Validation, Supervision, Project administration, Methodology, Investigation, Funding acquisition, Data curation, Conceptualization.

## Declaration of competing interest

The author is an Editorial Board Member/Editor-in-Chief/Associate Editor/Guest Editor for *Molecular Metabolism* and was not involved in the editorial review or the decision to publish this article.

The authors declare the following financial interests/personal relationships which may be considered as potential competing interests: PTP received speaker honoraria by Novo Nordisk. As a scientist, MHT participated in a scientific advisory board meeting of ERX Pharmaceuticals, Inc., Cambridge, MA, in 2019. He was a member of the Research Cluster Advisory Panel (ReCAP) of the Novo Nordisk Foundation between 2017 and 2019. He received funding for his research projects by Novo Nordisk (2016–2020) and Sanofi-Aventis (2012–2019). He consulted twice for Böhringer Ingelheim Pharma GmbH & Co. KG (2020 & 2021) and delivered a scientific lecture for Sanofi-Aventis Deutschland GmbH (2020) and for AstraZeneca GmbH (2024). As CEO and CSO of Helmholtz Munich, he is co-responsible for countless collaborations of the employees with a multitude of companies and institutions, worldwide. In this capacity, he discusses potential projects with and has signed/signs contracts for the centers institute(s) related to research collaborations worldwide, including but not limited to pharmaceutical corporations like Boehringer Ingelheim, Novo Nordisk, Roche Diagnostics, Arbormed, Eli Lilly, SCG Cell Therapy and others. As the CEO of Helmholtz Munich, he was/is further overall responsible for commercial technology transfer activities. MHT confirms that to the best of his knowledge none of the above funding sources or collaborations were involved in or had an influence on the preparation of this manuscript. All other authors declare that they have no conflict of interest related to this study.

## Data Availability

Data will be made available on request.
