# Supplementary material for: HDAC5 controls a hypothalamic STAT5b-TH axis, the sympathetic activation of ATP-consuming futile cycles and adult-onset obesity in male mice
Source: Mol Metab. 2024 Sep 19;90:102033. doi: 10.1016/j.molmet.2024.102033 (PMC11481749; doi:10.1016/j.molmet.2024.102033)
Supplement: Multimedia component 1 [file mmc1.pdf]

Supplemental Data

HDAC5 controls a hypothalamic STAT5b-TH axis, the sympathetic activation of ATP-consuming futile cycles and adult-onset obesity in male mice

Contreras Raian E.[1,2,3,4], Gruber Tim[2,3,5], González-García Ismael[2,3], Schriever Sonja C.[1,2,3], De Angelis Meri[2,3], Mallet Noemi[1,2,3], Bernecker Miriam[1,2,3,4], Legutko Beata[2,3], Kabra Dhiraj[1,2,3,6], Schmidt Mathias[7], Tschöp Matthias H.[8,9], Gutierrez-Aguilar Ruth[10,11], Mellor Jane[12,13], Garcia-Caceres Cristina[2,3,14] and Pfluger Paul T.[1,2,3,4,§]

[1]Research Unit NeuroBiology of Diabetes, Helmholtz Munich, Neuherberg, Germany; [2]Institute for Diabetes and Obesity, Helmholtz Munich, Neuherberg, Germany; [3]German Center for Diabetes Research (DZD), Neuherberg, Germany; [4]Neurobiology of Diabetes, TUM School of Medicine & Health, Technische Universität München, München, Germany; [5]Van Andel Institute, Grand Rapids, MI, USA; [6]Biological Research Pharmacology Department, Sun Pharma Advanced Research Company Ltd., Vadodara, India. [7]Neurobiology of Stress Resilience, Max Planck Institute of Psychiatry, Munich, Germany; [8]Division of Metabolic Diseases, TUM School of Medicine & Health, Technical University of München, Munich, Germany; [9]Helmholtz Center Munich, Neuherberg, Germany; [10]División de Investigación, Facultad de Medicina, Universidad Nacional Autónoma de México, Mexico City, Mexico; [11]Laboratorio de Investigación en Enfermedades Metabólicas, Obesidad y Diabetes, Hospital Infantil de México Federico Gomez, Mexico City, Mexico.[12]Department of Biochemistry, University of Oxford, Oxford, UK; [13]Chronos Therapeutics, Oxford, UK; [14]Medical Clinic and Polyclinic IV, Ludwig-Maximilians University of München, Munich, Germany.

Supplemental Figure 1

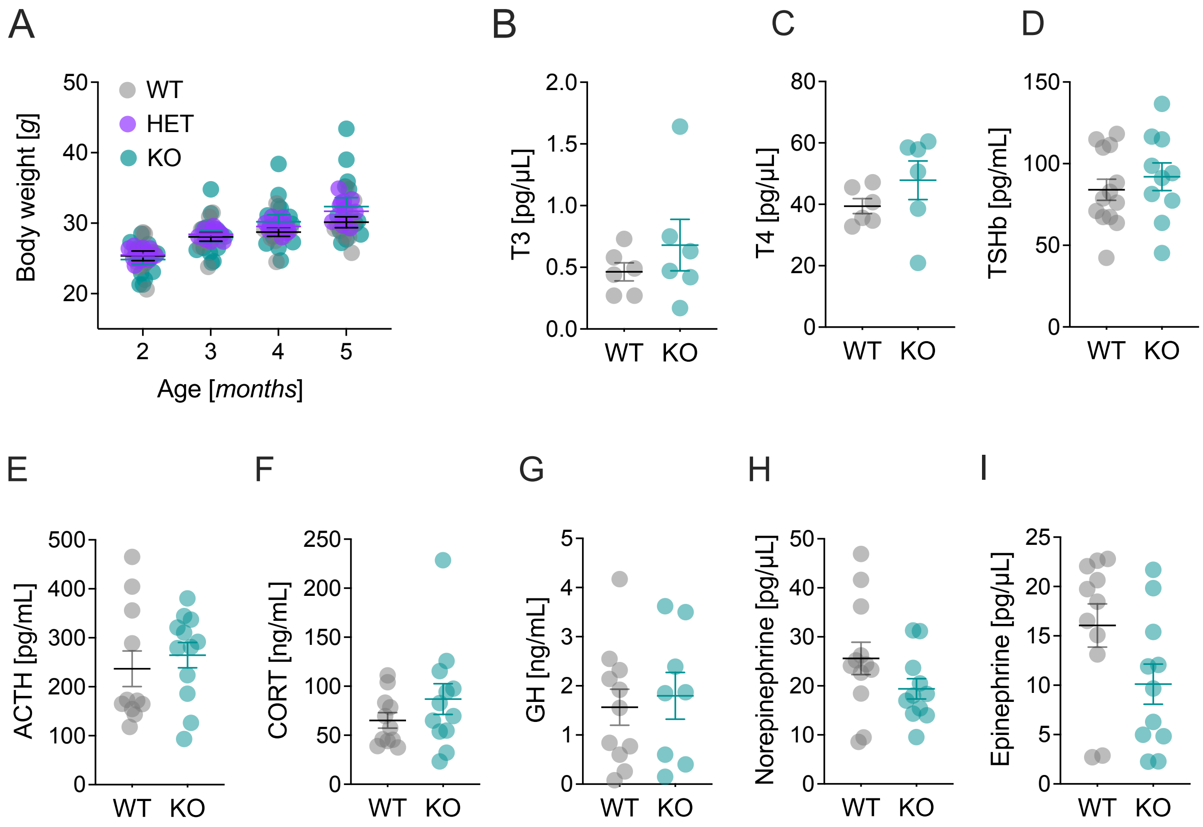

Supplemental Figure 1. Body weights and hormone levels in HDAC5 deficiency
(A) Chow-fed male WT, heterozygous (HET) and HDAC5-KO mice were evaluated for changes in body weight. Plasma from 6 months old HDAC5-KO and WT mice was used for quantifying (B-I) total triiodothyronine (T3) and (C) total thyroxine (T4), thyroid stimulating hormone subunit beta (TSHb), adrenocorticotropic hormone (ACTH), corticosterone (CORT), growth hormone (GH), norepinephrine and epinephrine. Values represent means ± SEM. Statistical analysis were done by or two-way ANOVA with Bonferroni post-hoc tests (B), or two-tailed unpaired Students' t-tests (B-I).

Supplemental Figure 2

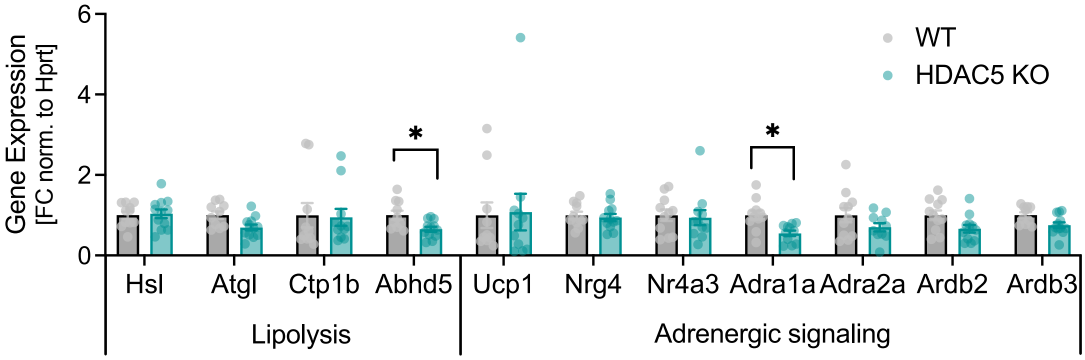

Supplemental Figure 2: Lipolytic and adrenergic gene expression changes in scWAT
Relative mRNA levels of key genes involved in lipolysis, including hormone-sensitive lipase (Hsl), adipose triglyceride lipase (Atgl), carnitine palmitoyltransferase 1B (Cpt1b) and alpha/beta-hydrolase domain-containing protein 5 (Abhd5), of uncoupling protein 1 (Ucp1), and of adrenergic receptor (signaling) genes neuregulin 4 (Nrg4), nuclear receptor subfamily 4 group A member 3 (Nr4a3), alpha-1A adrenergic receptor (Adra1a), alpha-2A adrenergic receptor (Adra2a), beta-2 adrenergic receptor (Ardb2) and beta-3 adrenergic receptor (Ardb3) in subcutaneous white adipose tissue (scWAT) of 6-months-old, chow fed male WT and HDAC5-KO mice. Values represent means ± SEM. Statistical analysis were done by two-tailed unpaired Students' t-tests. * $p < 0.05$.

Supplemental Figure 3

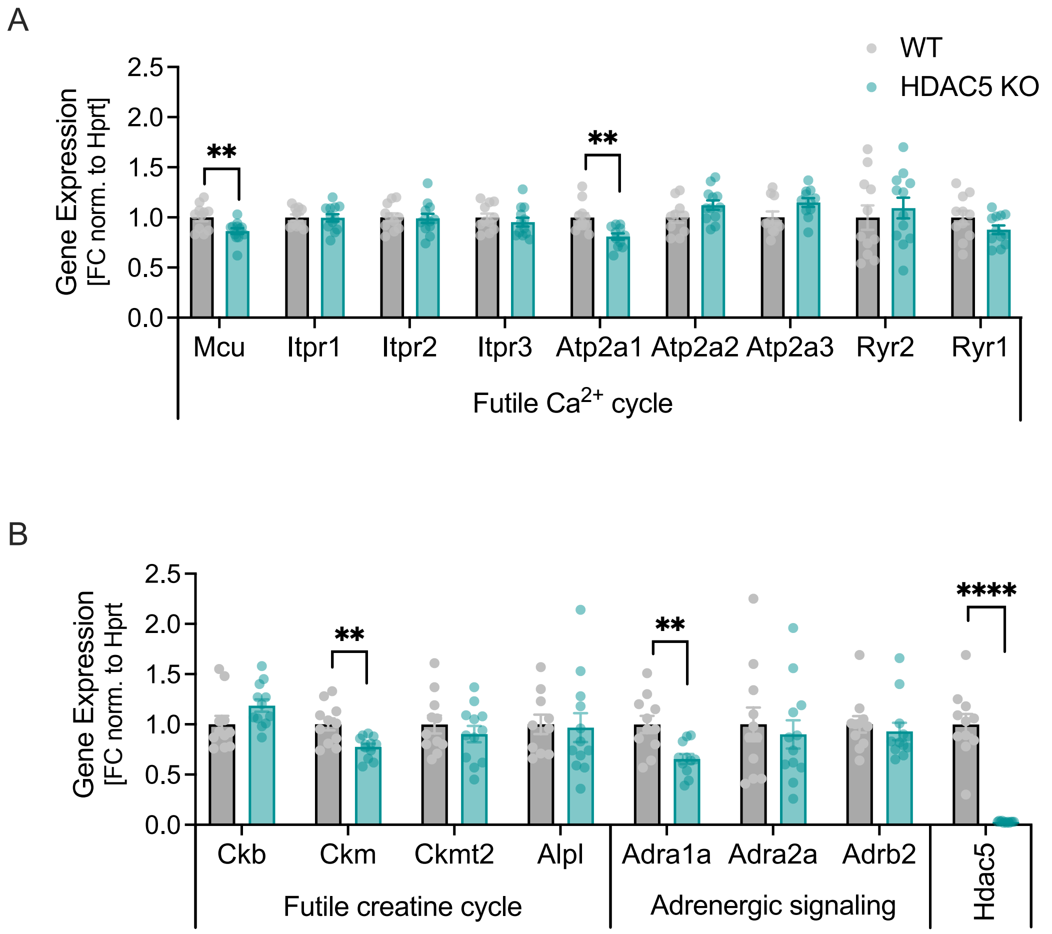

Supplemental Figure 3: Decreased expression levels of futile calcium and creatine cycle components in quadriceps skeletal muscles of 6-months-old chow fed male WT and HDAC5-KO mice.

(A) Relative mRNA levels of key genes involved in calcium cycling, including mitochondrial calcium uniporter (Mcu), inositol 1,4,5-trisphosphate receptor type 1 (Itpr1), inositol 1,4,5-trisphosphate receptor type 2 (Itpr2), inositol 1,4,5-trisphosphate receptor type 3 (Itpr3), ATPase sarcoplasmic/endoplasmic reticulum ca^{2+} transporting 1 (Atp2a1), ATPase sarcoplasmic/endoplasmic reticulum Ca^{2+} transporting 2 (Atp2a2), ATPase sarcoplasmic/endoplasmic reticulum Ca^{2+} transporting 3 (Atp2a3), ryanodine receptor 2 (Ryr2) and ryanodine receptor 1 (Ryr1). (B) Relative mRNA levels of genes involved in futile creatine cycling including creatine kinase B (Ckb), creatine kinase, muscle (Ckm), mitochondrial creatine kinase 2 (Ckmt2) and alkaline phosphatase, liver/bone/kidney (Alpl), as well as adrenergic receptors alpha-1A adrenergic receptor (Adra1a), alpha-2A adrenergic receptor (Adra2a), beta-2 adrenergic receptor (Adrb2), and Hdac5. Values represent means ± SEM. Statistical analysis were done by two-tailed unpaired Students' t-tests (A,B). ** $p < 0.01$ and **** $p < 0.0001$.

Supplemental Figure 4

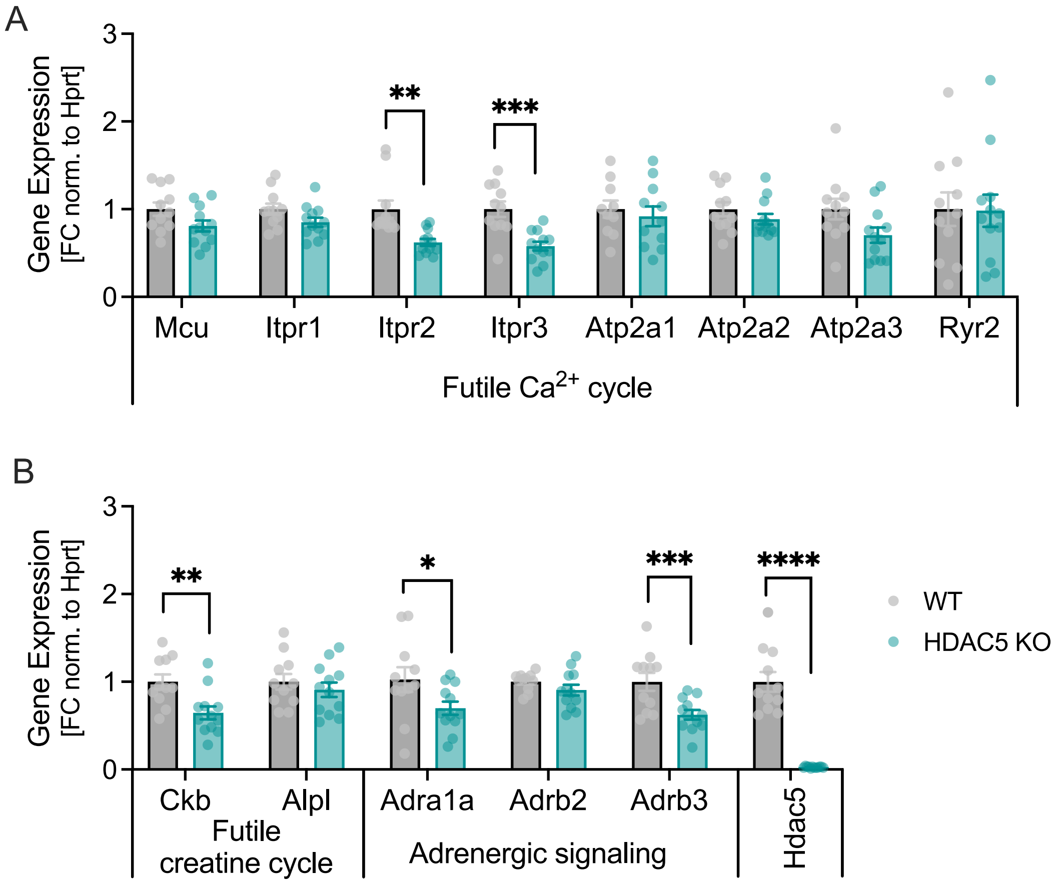

Supplemental Figure 4: Decreased expression levels of futile calcium and creatine cycle components in livers of 6-months-old chow fed male WT and HDAC5-KO mice. Relative mRNA levels of key genes involved in (A) calcium cycling, including Mcu, Itpr1, Itpr2, Itpr3, Atp2a1, Atp2a2, Atp2a3 and Ryr2, and of genes involved in (B) futile creatine cycling including Ckb and Alpl, as well as adrenergic receptors Adra1a, Adrb2 and Adrb3, and Hdac5. Values represent means ± SEM. Statistical analysis were done by two-tailed unpaired Students' t-tests (A,B). * $p < 0.05$, ** $p < 0.01$, *** $p < 0.001$ and **** $p < 0.0001$.

Supplemental Figure 5

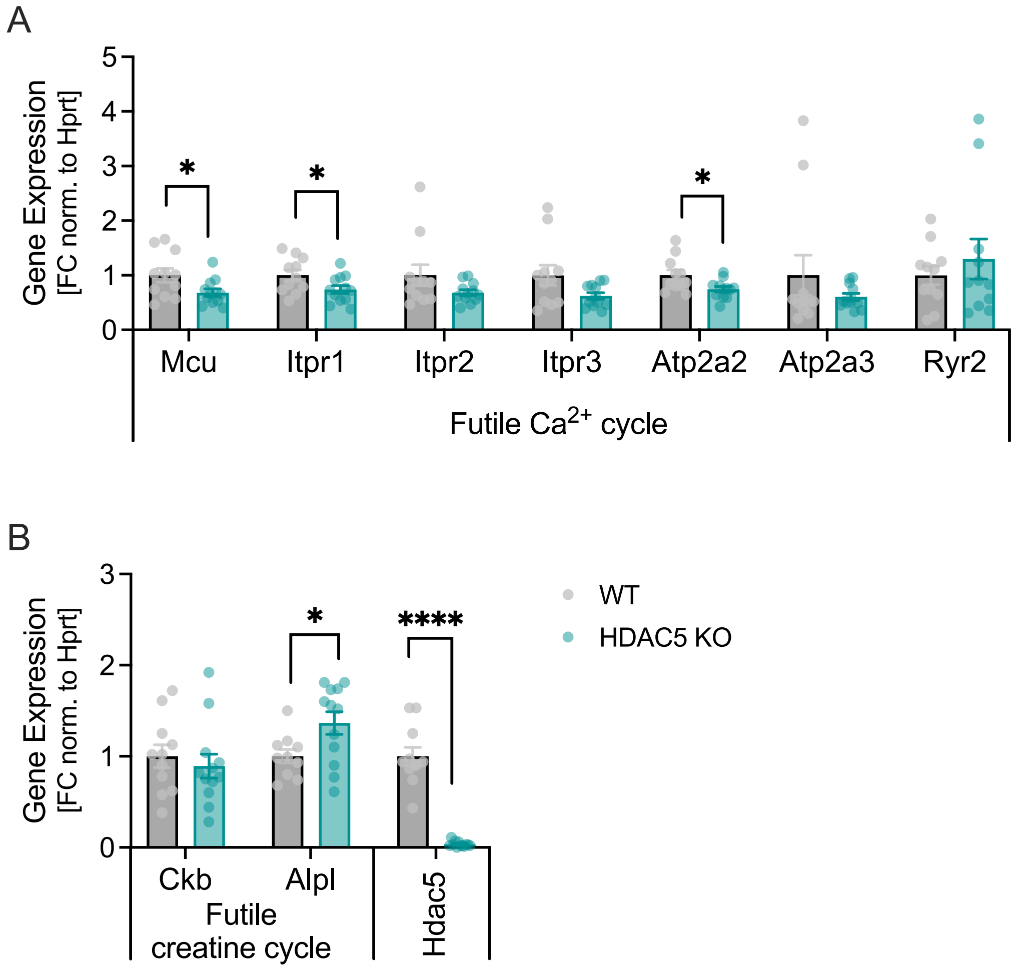

Supplemental Figure 5: Decreased expression levels of futile calcium and creatine cycle components in scWAT of 6-months-old chow fed male WT and HDAC5-KO mice. Relative mRNA levels of key genes involved in (A) calcium cycling, including Mcu, Itpr1, Itpr2, Itpr3, Atp2a2, Atp2a3 and Ryr2, and of genes involved in (B) futile creatine cycling including Ckb and Alpl, as well as Hdac5. Values represent means ± SEM. Statistical analysis were done by two-tailed unpaired Students' t-tests (A,B). * $p < 0.05$ and **** $p < 0.0001$.

Supplemental Figure 6

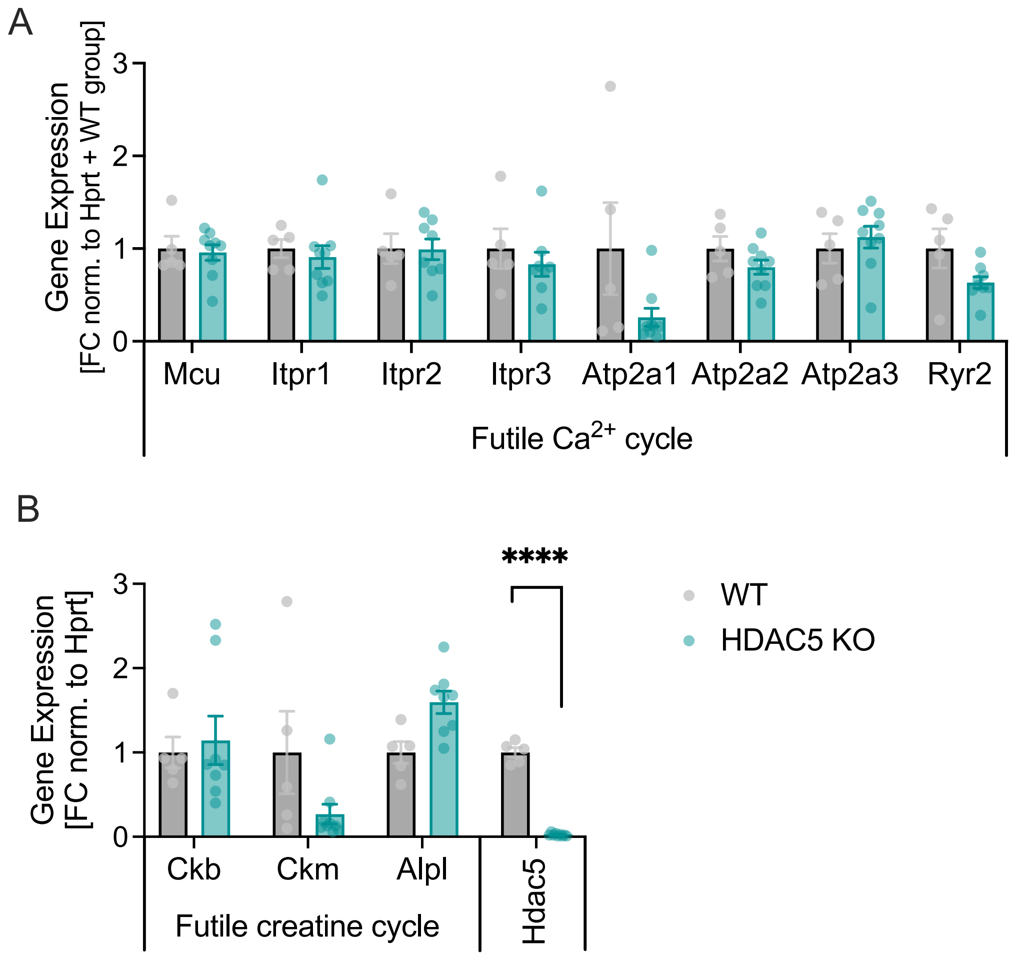

Supplemental Figure 6: Unperturbed expression levels of futile calcium and creatine cycle components in eWAT of 6-months-old chow fed male WT and HDAC5-KO mice. Relative mRNA levels of key genes involved in (A) calcium cycling and (B) futile creatine cycling. Values represent means ± SEM. Statistical analysis were done by two-tailed unpaired Students' t-tests (A,B).

Supplemental Figure 7

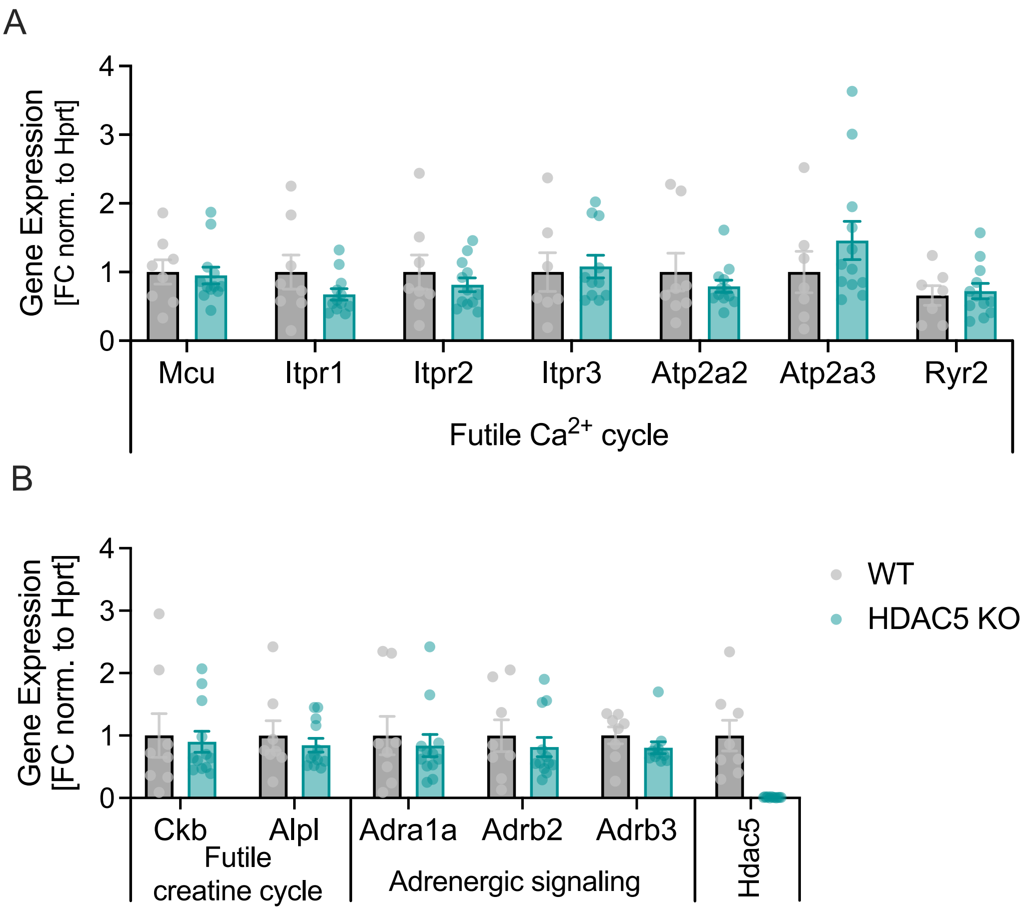

Supplemental Figure 7: Unperturbed expression levels of futile calcium and creatine cycle components in BAT of 6-months-old chow fed male WT and HDAC5-KO mice. Relative mRNA levels of key genes involved in (A) calcium cycling and (B) futile creatine cycling. Values represent means ± SEM. Statistical analysis were done by two-tailed unpaired Students' t-tests (A,B).

Supplemental Figure 8

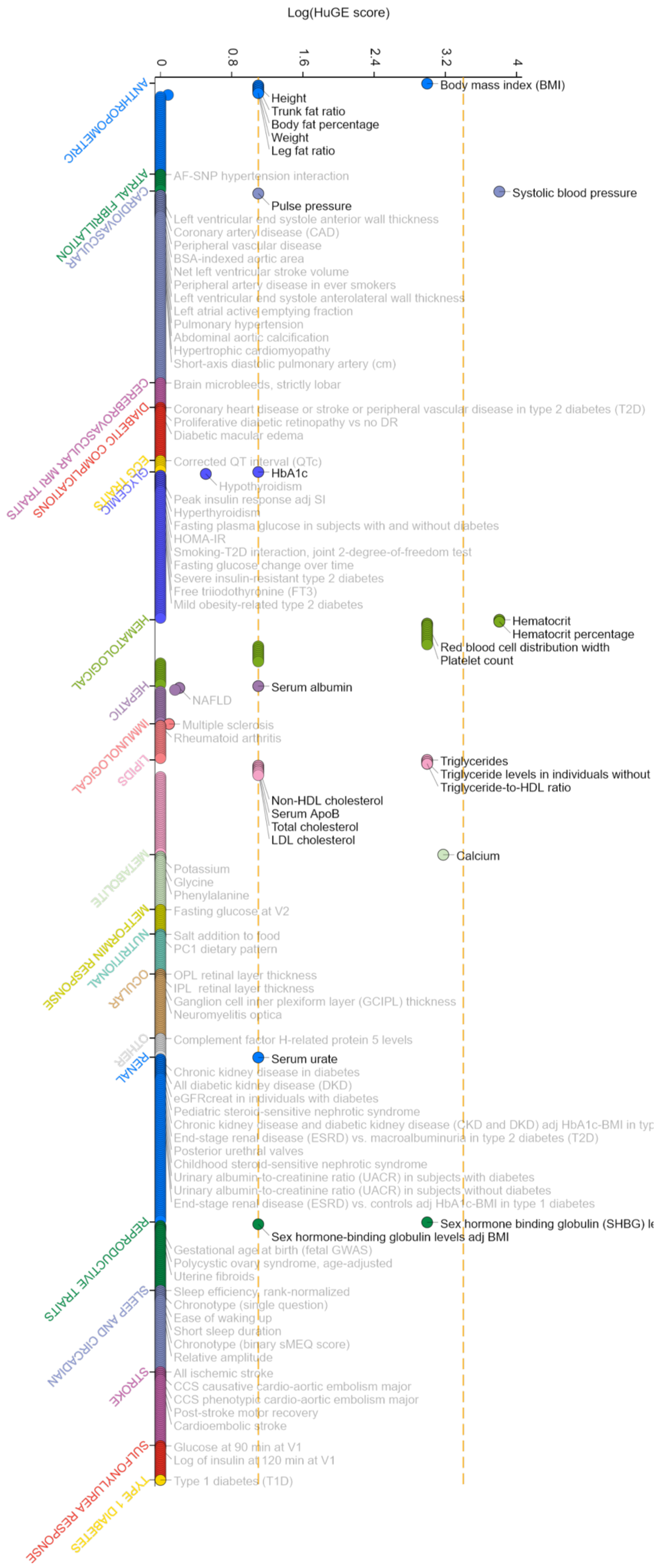

Supplemental Figure 8. Human genetic evidence score for HDAC5. Human genetic evidence score for HDAC5. This score is based on genetic and genomic data by analyzing the association of common and rare variants with a specific phenotype. Values above 3.2 of the log score (upper punctuated line) indicate very strong evidence that HDAC5 is involved in a specific phenotype. Between the two punctuated lines, the score represents strong to moderate evidence.

Supplemental Tables

Supplemental Table 1. Master Mix for HDAC5-KO mouse genotyping.

Master Mix	Concentration	Volume (μL)
MilliQ H_20		7,18
Rx buffer	5X	3,00
MgCl2	25mM	1,20
dNTP	10mM	0,30
HD5 gt 5' primer	20μM	0,60
HD5 gt low 3'-1	20μM	0,30
LacZ 3'-2	20μM	0,30
Promega Go-Taq flexi	5U/μl	0,12
cDNA		2

Supplemental Table 2. PCR protocol for HDAC5-KO mice.

Step	Temperature [°C]	Time	N° cycles
Initial denaturation	95	4 min	1
Denaturation	95	30 s	
Annealing	62	30 s	35
Elongation	72	45 min	
Final elongation	72	5 min	1
Hold	4	∞	

Supplemental Table 3. Genotyping primers.

Primers ID	5'-Primer sequences-3'
HDAC5-KO	
LacZ 3’2:	GCCAGTTTGAGGGGACGACGACAGTATCG
HD5 gt 5’:	CAAGGCCTTGTGCATGCTGGGCTGG
HD5 gt low 3’1:	CTGCTCCCGTAGCGCAGGGTCCATG

Supplemental Table 4. Taqman assays for selected genes.

Gene name	Taqman assay
Adr2b	Mm02524224_s1
Adr3b	Mm02601819_g1
Adra2a	Mm07295458_s1
Agrp	Mm00475829_g1
Drd2	Mm00438545_m1
Fasn	Mm00662318_g1
Gh	Mm00433590_g1
Ghsr	Mm00616415_m1
Gfap	Mm01253033_m1
Hdac5	Mm01246076_m1
Hprt	Mm01545399_m1
Iba1	Mm00479862_g1
Lpl	Mm00434764_m1
Malat1	Mm01227912_s1
Pomc	Mm00435874_m1
Ppargc1a	Mm01208835_m1
Prdm16	Mm00712556_m1
Rbfox3	Mm01248771_m1
Slc6a3	Mm00438388_m1
Th	Mm00447557_m1
Ucp1	Mm01244861_m1

Supplemental Table 5. SYBR-green primer pairs for selected genes.

Gene name	Primer	5‘-Primer Sequences-3‘
Abhd5	Forward	TGGTGTCCCACATCTACATCA
	Reverse	CAGCGTCCATATTCTGTTTCCA
Acc	Forward	CTAGTAGCTCTTACTTCCGGGAC
	Reverse	GAAACTGGTCTCCCATGTTCAT
Adra1a	Forward	CTAAGGCCATTCTACTTGGGGT
	Reverse	CGAGTGCAGATGCCGATGA
Adra2a	Forward	GTGACACTGACGCTGGTTTG
	Reverse	CCAGTAACCCATAACCTCGTTG
Adrb2	Forward	ATGGGGCCACACGGGAACGA
	Reverse	GTGACGTGGTCTGGCGC
Adrb3	Forward	AAGCCCTCAGCATCCCAAGCTA
	Reverse	CCTGGCAGAACCTGAGGCAACC
Alpl	Forward	CCAACTCTTTTGTGCCAGAGA
	Reverse	GGCTACATTGGTGTTGAGCTTTT
Atgl	Forward	CACAGCGCTGGTCACTGGGG
	Reverse	CCGGGCCTCCTTGGACACCT
Atp2a1	Forward	TGTTTGTCCTATTTCGGGGTG
	Reverse	AATCCGCACAAGCAGGTCTTC
Atp2a2	Forward	TGGAACAACCCGGTAAAGAGT
	Reverse	CACCAGGGGCATAATGAGCAG
Atp2a3	Forward	GGAGCAGTTTGAGGACCTCTT
	Reverse	GGCCACGAGAATTAGCATGATG
Ckb	Forward	AGTTCCCTGATCTGAGCAGC
	Reverse	GAATGGCGTCGTCCAAAGTAA
Ckm	Forward	CTGACCCCTGACCTCTACAAT
	Reverse	CATGGCGGTCCTGGATGAT
Ckmt2	Forward	ACACCCAGTGGCTATACCCTG
	Reverse	CCGTAGGATGCTTCATCACCC
Cpt1b	Forward	TGGCTACGGGGTCTCTTACA
	Reverse	GGGCGTTCGTCTCTGAACT T
Fasn	Forward	AGAGATCCCGAGACGCTTCT
	Reverse	GCTTGGTCCTTTGAAGTCGAAGA
Gad1	Forward	CACAGGTCACCCTCGATTTTT
	Reverse	ACCATCCAACGATCTCTCTCATC
Gad2	Forward	TCCGGCTTTTGGTCCTTCG
	Reverse	ATGCCGCCCGTGAACTTTT

Hsl	Forward	CCGTTCCTGCAGACTCTCTC
	Reverse	CCACGCAACTCTGGGTCTAT
Itpr1	Forward	CGTTTTGAGTTTGAAGGCGTTT
	Reverse	CATCTTGCGCCAATTCCCG
Itpr2	Forward	TTCAGTTCCTATCGAGAGGATGT
	Reverse	GCTGATTGACGCAAGGTCG
Itpr3	Forward	GGGCGCAGAACAACGAGAT
	Reverse	GAAGTTTTGCAGGTCACGGTT
Mcu	Forward	GAGCCGCATATTGCAGTACG
	Reverse	CGAGAGGGTAGCCTCACAGAT
Nr4a3	Forward	TGGACAAGAGACGCCGAAAC
	Reverse	TGGTTTGGAAGGCAGACGAC
Nrg4	Forward	CACGCTGCGAAGAGGTTTTTC
	Reverse	CGCGATGGTAAGAGTGAGGA
Oxtr	Forward	GATCACGCTCGCCGTCTAC
	Reverse	CCGTCTTGAGTCGCAGATTC
Ppara	Forward	TACTGCCGTTTTCACAAGTGC
	Reverse	AGGTCGTGTTCACAGGTAAGA
Pparg	Forward	CCAGAGCATGGTGCCTTCGCT
	Reverse	CAGCAACCATTGGGTCAG
Prl	Forward	CAGGGGTCAGCCCAGAAAG
	Reverse	TCACCAGCGGAACAGATTGG
Prlr	Forward	CCACATTCCTGTGCTCATCCT
	Reverse	AGTCTGACTTACATGGTGTCCA
Ryr1	Forward	CAGTTTTTGCGGACGGATGAT
	Reverse	CACCGGCCTCCACAGTATTG
Ryr2	Forward	ACGGCGACCATCCACAAAG
	Reverse	AAAGTCTGTTGCCAAATCCTTCT
Tshb	Forward	TGGATAGGAGAGAGTGTGCC
	Reverse	GTGTCATACAATACCCAGCACAG
Ucp1	Forward	AGGCTTCCAGTACCATTAGGT
	Reverse	CTGAGTGAGGCAAAGCTGATTT

Supplemental Material & Reagents

Reagents

- µMACS G-protein MicroBeads, 130 071 101, Miltenyi Biotec.
- AccuGene 0.5 EDTA, 51201, SZABO SCANDI.
- AMPure XP beads, A63882, Beckman Coulter.
- Bovine Serum Albumin (BSA), 422361V, VWR.
- Clarity™ ECL substrate, 1705060, Bio-Rad
- Criterion™ TGX™ Precast Gels, 5671093, Bio-Rad.
- DABCO, 0718m, Carl ROTH.
- Dithriothreitol (DTT), R0861, Thermo Fischer.
- D(+)-Saccharose, 4621.2, Carl ROTH.
- Dulbecco's Modified Eagle's Medium DMEM, 11880-028, Sigma-Aldrich
- Gelatin, Carl ROTH.
- Glycine, G7126-100G, Sigma-Aldrich.
- HALT™ (100X), phosphatase and protease inhibitor, 78429, Thermo Fischer.
- IGEPAL® CA-630, 18896-50ML, Sigma-Aldrich.
- Isoproterenol hydrochloride, 16504-100MG, Sigma-Aldrich.
- KCL (2M), AM9640G, Thermo Fisher.
- Methanol ≥99.9%, EMSURE® ACS, 1.06009.2500, VWR
- MgCl2 (1M), AM9530G, Thermo Fisher.
- Mowiol 4-88, 0713, Carl ROTH.
- NaCl, 9265.1, Carl ROTH.
- NuPAGE™ LDS sample buffer, NP0008, Thermo Fischer.
- O.C.T, 4583, Tissue-TeK, Alphen aan den Rijn.
- PBS pH 7.4, 10010, Thermo Fischer.

- Penicillin-Streptomycin (PenStrep), 15140-122, Gibco / ThermoFischer.
- Phenyl-methane-sulfonylfluorid (PMSF), Carl ROTH.
- Restore™ Plus stripping buffer, 46430, Thermo Fischer.
- RNasin® Plus, N2111, Promega.
- Spermidine trihydrochloride, 85580-5G, Sigma-Aldrich.
- Spermine, S3256-1G, Sigma-Aldrich.
- SUPERase In™ (20U/µL), AM2694, Thermo Fischer.
- Trans-Blot Turbo 0.2µm PVDF Transfer Packs, 170-4157, Bio-Rad.
- Trans-Blot Turbo 0.2µm Nitrocellulose Transfer Packs, 170-4159, Bio-Rad.
- Triton X-100, T8532, Sigma-Aldrich.
- Tris (1M) pH 8, AM9855G, Thermo Fisher.
- Tris PUFFERAN® ≥99,9% p.a., 4855.2, Carl ROTH.
- Tween 20, P1379-500ML, Sigma-Aldrich.

Materials

- Large clearance Pestle A, 885301-0007, Kimble Chase.
- Large clearance Pestle B, 885302-0007, Kimble Chase.
- Low Protein binding collection tubes 1.5mL, 90410, Thermo Fischer.
- PluriStrainer mini 10µm, 43-10010-40, PluriSelect.
- SDS pellets, CN30.2, Carl ROTH.
- Tissue grinder (douncer) 7mL, 885303-0007, Kimble Chase.

Commercial kits

- Agilent RNA 6000 Pico kit, 5067-1513, Agilent.

- Free glycerol colorimetric assay kit, K630-100, Quimigen.
- Rat/Mouse growth hormone ELISA kit, EZRMGH-45K, Merk.
- Ultra-sensitive mouse insulin ELISA kit, 90082, Crystal Chem.
- Rat/Mouse leptin quantikine ELISA kit, MOB00, R&D Systems.
- LabAssay™ Cholesterol, Wako, FUJIFILM.
- LabAssay™ Tricylglyceride, Wako, FUJIFILM.
- Non-esterified fatty acids, NEFA-HR(2) Assay, Wako, FUJIFILM.
- Pierce™ BCA Protein Assay Kit, 23225, Thermo Fischer.
- Prolactin mouse ELISA, EMPRL, Life Technologies
- QuantiTect® Reverse Transcription Kit, 205311, QIAGEN.
- RNeasy Micro Kit, 74004, QIAGEN.
- SMART-Seq® v4 Ultra® Low Input RNA kit, 634888, Takara Bio USA, Inc.
- Tshb mouse ELISA, USC-CEA463MU-96, Biozol diagnostica.

Antibodies

- α-Tubulin, T5168-100UL, Sigma-Aldrich.
- β-Actin (C4), sc-47778, Santa Cruz Biotechnology.
- cFOS, 226-003, Synaptic Systems.
- Donkey anti-rabbit IgG-Alexa Fluor 568, A10042, Thermo Fischer.
- Donkey anti-mouse IgG-Alexa Fluor 488, A21202, Thermo Fischer.
- Donkey anti-goat IgG-Alexa Fluor 647, A21447, Thermo Fischer
- Donkey-anti-goat IgG-HRP, sc-2020, Santa Cruz Biotechnology.
- GAPDH (D6), sc-166545, Santa Cruz Biotechnology.
- Goat anti-rabbit IgG-HRP, A16096, Life Technologies.
- Goat-anti-mouse IgG-HRP, sc2005, Santa Cruz Biotechnology.

- HDAC5 (B-11), sc133106, Santa Cruz Biotechnology.
- Histone 3 (H3), ab1791, Abcam.
- HSL, 4107S, Cell Signaling.
- Mouse (G3A1) IgG1-isotype control, 5415S, Cell Signaling.
- NeuN-conjugated Alexa fluor® 488, MAB377X, Sigma-Aldrich.
- UCP1, ab10983, Abcam.
- p44/42 MAPK (Erk1/2), 9102S, Cell Signaling.
- Phospho-p44/42 MAPK (Erk1/2) (Thr202/Tyr204), 9101S, Cell Signaling.
- Phospho-HSL (Ser660), 4126S, Cell Signaling.
- Phospho-STAT5 (Tyr694), 9351S, Cell Signaling
- STAT5, 9363S, Cell Signaling.
- STAT5b, 13-5300, Thermo Fischer.
- Tyrosine hydroxylase, AB1542, Sigma-Aldrich.

Instruments

- EcoMRI, E26-217M, EchoMRI.
- FreeStyle™ Blood Glucometer, Abbot.
- FreeStyle™ Lite Blood Glucose Test strips, 70814-70, Abbot.
- Infrared camera PI450i, Optris Infrared measurements.
- Leica CM3050 Cryostat, Leica Biosystems.
- Leica TCS SP5, Leica Biosystems.
- NanoDrop 2000 Spectophotometer, Thermo Fischer.
- Perfusion pump, P720/66, Instech Laboratories.
- Tissue Lyser II, QIAGEN.
- Trans-Blot® Turbo™ Transfer system, Bio-Rad.

- Ultrasonic homogenizer, model 150V/T, Biologics Inc.
- Viia7 cycler, Applied Biosystems, Thermo Fischer.
